# Immunoglobulin Kappa Light Chain Produced by Cardiomyocytes and Participates in Maintaining Intercalated Disc Integrity

**DOI:** 10.1002/mco2.70940

**Published:** 2026-09-09

**Authors:** Zhu Zhu, Yixuan Sheng, Yun Chang, Yuan Chang, Miaomiao Xu, Wenjing Zhou, Wenxiu Wang, Qingyuan Liao, Zhan Shi, Meng Zhang, Feng Lan, Jiangping Song, Xiaoyan Qiu

**Affiliations:** ^1^ NHC Key Laboratory of Medical Immunology Peking University Beijing China; ^2^ Department of Immunology School of Basic Medical Sciences Beijing China; ^3^ State Key Laboratory of Cardiovascular Disease Fuwai Hospital National Center for Cardiovascular Diseases Chinese Academy of Medical Sciences and Peking Union Medical College Beijing China; ^4^ State Key Laboratory of Animal Biotech Breeding College of Biological Sciences China Agricultural University Beijing China; ^5^ Fuwai Hospital Chinese Academy of Medical Sciences Shenzhen Key Laboratory of Cardiovascular Disease State Key Laboratory of Cardiovascular Disease Key Laboratory of Pluripotent Stem Cells in Cardiac Repair and Regeneration Chinese Academy of Medical Sciences and Peking Union Medical College Shenzhen China; ^6^ Department of Cardiac Surgery Fuwai Hospital National Center for Cardiovascular Diseases Chinese Academy of Medical Sciences and Peking Union Medical College Beijing China; ^7^ Beijing Key Laboratory of Xenotransplantation Fuwai Hospital National Centre for Cardiovascular Disease Chinese Academy of Medical Sciences and Peking Unionmedical College Beijing China; ^8^ Department of Epidemiology and Biostatistics School of Public Health Peking University Beijing China; ^9^ Department of Immunology Guilin Medical University Guangxi China; ^10^ Department of Cardiology Aerospace Center Hospital Beijing China; ^11^ Clinical Research Institute Chinese Academy of Medical Sciences Faculty of Clinical Medicine Peking Union Medical College Beijing China

**Keywords:** cardiac contraction, desmoplakin, immunoglobulin kappa light chain (Igκ), intercalated disc, plectin

## Abstract

In this study, we unexpectedly found that the immunoglobulin kappa light chain (Igκ) was expressed in normal cardiomyocytes of mice and humans, especially in intercalated discs (ICDs). Cardiomyocyte‐specific knockout of Igκ in mice results in reduced myocardial contraction, atrioventricular block (AV block), and even sudden death. Histological analysis revealed that Igκ knockout in cardiomyocytes leads to structural disorder of ICDs, dissemination of the cytoskeleton proteins desmin and F‐actin, as well as the loss of desmoplakin (DSP), N‐cadherin, and connexin 43 (Cx43) on ICDs. Mechanistically, Igκ can bind to and stabilize plectin, a cytoskeleton‐cross‐linking protein that facilitates the assembly of desmin‒actin networks that maintain normal cytoskeletal architecture. Igκ can also anchor desmin to the DSP through plectin, thereby stabilizing the integrity of the ICDs. This molecular interplay critically reinforces cardiomyocyte cohesion and maintains structural and functional homeostasises. Our findings are the first to identify cardiomyocyte‐expressed Igκ as a novel ICD‐related molecule that participates in cardiomyocyte contraction and conduction by stabilizing plectins. Importantly, this work extends current arrhythmogenic cardiomyopathy (ACM) pathogenic models by revealing that ablation of the non‐desmosomal gene Igκ disrupts ICD integrity, uncovering a new mechanism for non‐desmosomal gene‐related ACM and highlighting Igκ as a potential target for therapeutic investigations.

## Graphical abstract

This study identifies Igκ expression in cardiomyocytes, with predominant localization at ICDs. Cardiomyocyte‐specific Igκ knockout induces cardiac dysfunction and ICDs structural disorganization. Mechanistically, Igκ stabilizes plectin to facilitate desmin‒actin network assembly and anchor desmin to DSP, which is critical for maintaining ICDs structural integrity.

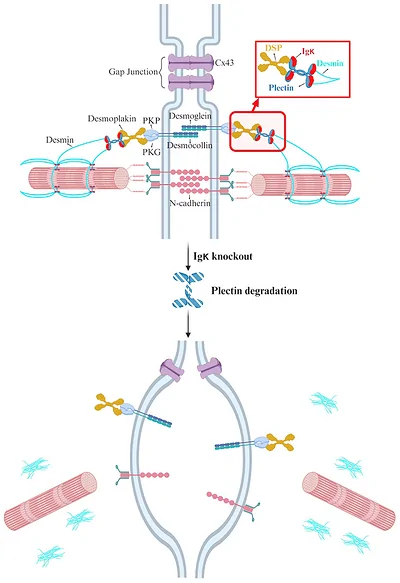

## 1 Introduction

Immunoglobulins (Igs) are key components of the immune system, and are primarily known for their role as antibodies. Traditionally, B cells and plasma cells that differentiate from B cells have been regarded as the sole producers of Igs. However, recent research has demonstrated that Igs are widely expressed in non‐B‐cell lineages (non‐B Igs), including epithelial cells, neurons, and testicular germ cells [1, 2, 3, 4, 5, 6, 7, 8]. Beyond their function as natural antibodies in immune defense, non‐B Igs exhibit diverse biological roles. For instance, IgG derived from renal podocytes supports cell adhesion and survival [9, 10], free Igκ from hepatocytes participates in regulating hepatocyte survival [11], and cancer‐derived IgG plays a role in the survival, adhesion, migration, and metastasis of cancer cells by forming focal adhesions through integrin binding [12, 13, 14]. These findings indicate that Igs are produced by various cell types and that their functions are not merely to act as antibodies, but also to exert multiple physiological and pathological functions.

Clinical observations have linked increased Ig levels to heart disease. For example, IgE levels rise sharply during acute myocardial infarction [15], local IgM deposition occurs in injured cardiomyocytes (CMs) [16, 17], and serum levels of Ig free light chains (FLCs) are frequently elevated in patients with heart failure, suggesting their potential role as novel prognostic markers [18]. Notably, the deposition of Ig light chains in heart tissue affects both the mechanical and structural properties of the heart and exerts direct cytotoxicity on CMs, serving as a pathogenic marker of irreversible myocardial amyloidosis [19, 20, 21].

Despite extensive studies on the role of Igs in heart disease, the prevailing assumption is that they originate exclusively from the B cells. Given our findings and those of others indicating that non‐B cells can produce Igs, we investigated whether CMs could also synthesize these molecules. In 2009, Mehta and coworkers reported the presence of Ig heavy and light chain transcripts in mouse heart tissue and HL‐1 myocardial cells [22]. In 2017, Our study further demonstrated that IgM heavy chains are present in CMs from both wild‐type (WT) and B‐cell‐deficient mice, with ischemia and hypoxia enhancing IgM expression and secretion [23]. These studies suggest that CMs can produce Igs, warranting further exploration of the physiological roles of CM‐derived Igs (CM‐Igs).

Classical Igs consist of two identical heavy chains and two identical light chains, with five types of heavy chains (Igμ, Igγ, Igα, Igδ, and Igε) and two types of light chains (κ and λ). Although each light chain can pair with any heavy chain, their frequencies differ by a ratio of 20:1 for κ to λ in mice. Our previous research revealed that CMs contain IgM, IgG, and IgA, with Igκ as the primary light chain primarily located at the intercalated discs (ICDs) of these cells. Therefore, this study focused on the expression of Igκ in CMs and its physiological functions.

We have demonstrated that CMs can synthesize Igκ, and mice with CM‐specific Igκ deficiency exhibit phenotypes including electrophysiological abnormalities, impaired myocardial contraction function, and sudden death. Notably, replenishing Igκ with adeno‐associated virus (AAV) therapy completely rescues these symptoms completely. This study provides the first evidence linking CM‐derived Igκ to cardiac contraction and conduction functions. Furthermore, we demonstrate that Igκ knockout promotes degradation of the cytoskeletal scaffold protein plectin, thereby destabilizing the component of the ICDs. This disruption leads to disintegration of the normal cytoskeletal architecture and ultimately resulting in CM dysfunction.

## 2 Results

### 2.1 Igκ Can be Produced by the CMs and Localized on ICDs

**FIGURE 1:**
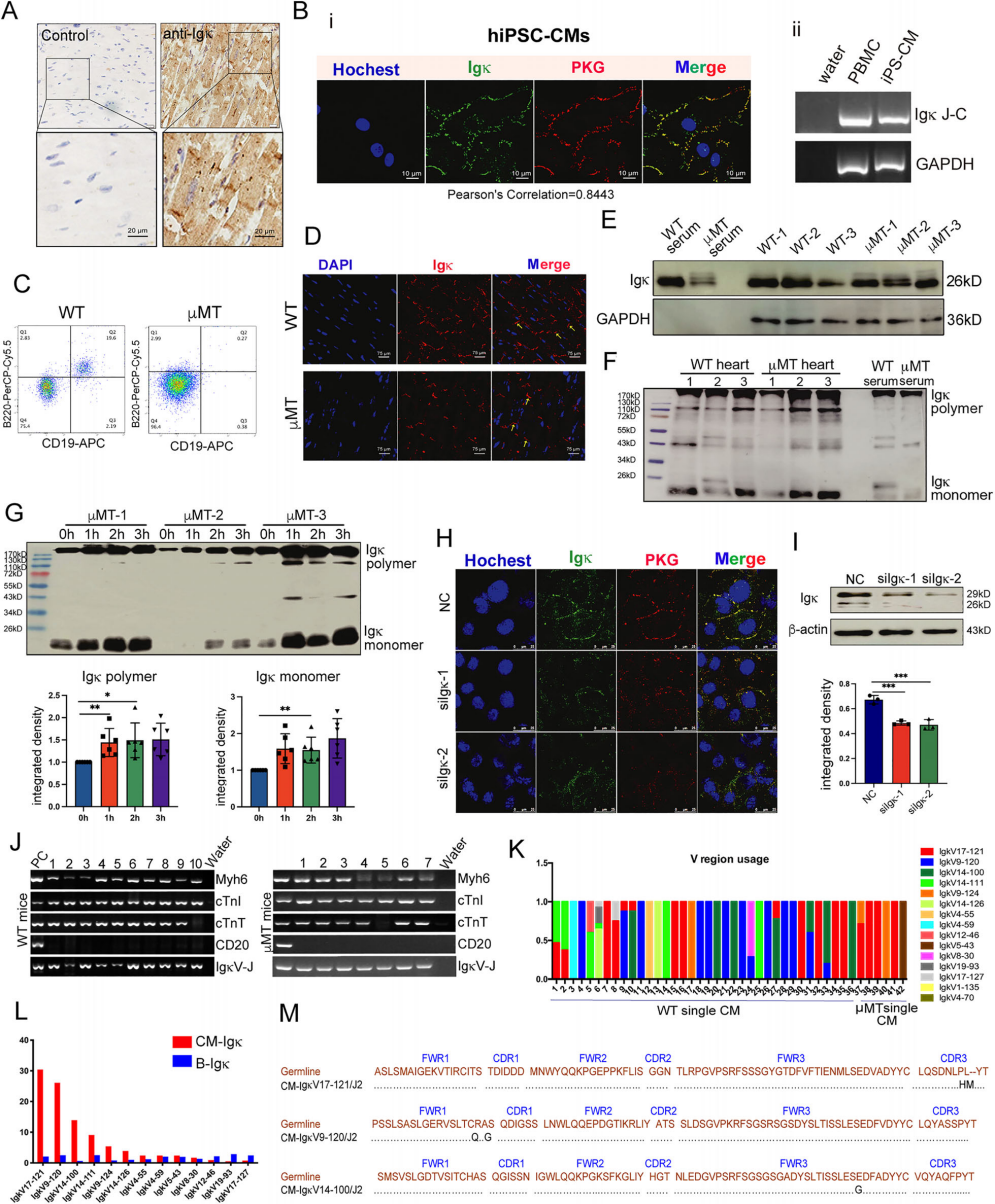
Igκ is expressed in cardiomyocytes (CMs) of mice and human with restricted VJ diversity. (A) Immunoglobulin kappa light chain (Igκ) staining was detected on the CM of human heart autopsy specimen by immunohistochemistry (IHC). Scale bar = 20 µm. Section without primary antibody was used as control. (B) (i) Igκ staining was found to be colocalized with plakoglobin (PKG) on the cell membrane of hiPSC‐CMs. Pearson's correlation = 0.8443. Scale bar = 10 µm. (ii) Igκ was detected in hiPSC‐CM by RT‐PCR. (C) B cells were absent in peripheral blood of µMT mice by FACS. (D) Igκ staining (red) was detected mainly on the intercalated discs (ICDs) in µMT and wild‐type (WT) heart tissue by immunofluorescence. Scale bar = 75 µm. (E) Igκ expression was detected in heart tissue and serum of both µMT and WT mice by Western blot analysis under reduced condition, GAPDH was used as an internal control. (F) Free Igκ with monomer and polymer was detected in both WT and µMT mice heart tissue under non‐reduced condition. µMT indicates B‐cell‐deficient mice. (G) µMT heart was removed and cultured in 500 µL DMEM without FBS for 3 h, 20 µL supernatant was collected and detected for Igκ under non‐reduced condition per hour. Statistical analysis of the integrated density of Igκ was performed with RM one‐way ANOVA, followed by Tukey's multiple comparisons test. ^*^ *p *< 0.05, ^**^ *p* < 0.01, ^***^ *p* < 0.001, ^****^ *p* < 0.0001. (H) Igκ staining (green) was detected in the cell junction of HL‐1 cells, and the positive signal was diminished by using two specific siRNA for Igκ. Scale bar = 25 µm. PKG (red) was used as marker of cell junction. NC indicates negative control. (I) Igκ knockdown on protein levels in HL‐1 cells by two independent siRNA. β‐Actin was used as an internal control. The protein level of Igκ was normalized to β‐actin loading control. Quantitative data are shown as means ± SDs. Statistical analysis was performed with one‐way ANOVA with Tukey's multiple comparisons. ^*^ *p* < 0.05, ^**^ *p* < 0.01, ^***^ *p* < 0.001, ^****^ *p* < 0.0001. (J) Igκ transcript was detected in single CM of WT and µMT mice by RT‐PCR. Myh6, cTnI, and cTnT were used as CM marker and heart tissue of WT mice was used as positive control (PC). CD20 was used as B‐cell marker and spleen of WT mice was used as PC. (K) V segment used in single CM (*n* = 42), 36 of WT single CMs (Nos. 1‒36) and six of µMT single CMs (Nos. 37‒42) were analyzed by Sanger sequencing. (L) The frequency of Vκ gene segments used by CM compared with B cells by Sanger sequencing and testified by the next‐generation sequencing. B, B cells. (M) Sequence of Igκ variable region in single CM.

To assess Igκ expression in CMs, immunohistochemical (IHC) staining was initially performed on human autopsy cardiac specimens. The results revealed that Igκ was distributed in CMs, including on the cell membrane, sarcomeres, and ICDs, with the strongest fluorescent signal detected at the ICDs (Figure 1A). Given the rapid occurrence of high levels of FLC in circulation during acute heart failure, and the fact that the heart is a commonly affected by organ due to hydrophobic deposition of FLC, we hypothesized that myocardial cells can express Igκ. Thus, we use human‐induced pluripotent stem cell‐derived CM (hiPSC‐CM) to eliminate the contamination of other cells and verified the presence of Igκ on the cell membrane of hiPSC‐CM, which colocalized with plakoglobin (PKG), with a colocalization coefficient of 0.8443 (Figures 1B and S1A,B). Subsequently, to further verify Igκ synthesis by CMs, IHC was performed to detect Igκ type light chain in cardiac tissues from B‐cell‐deficient (µMT) (Figure 1C) and WT mice. Comparable Igκ staining intensity at ICDs was observed between the µMT and WT mice (Figures 1D and S1C,D). Next, we found that the Igκ type LC with monomer, dimer, and polymers, was detected in CMs of µMT and WT mice by non‐reduced SDS‒PAGE/Western blot, indicating that Igκ produced by CMs exists in free form (κFLC) (Figures 1E,F). Moreover, κFLC is secreted into the extracellular space during ischemia and hypoxia (Figure 1G). To exclude the contamination by non‐CMs, we also identified Igκ in HL‐1 myocardial cells and primary cultured CMs from neonatal mice. Interestingly, in HL‐1 cells, Igκ was localized at cell junctions marked by PKG (Figure 1H), while in primary cultured CMs of neonatal mice, it was observed in cross‐striations (Figure S1E). Knockdown of Igκ via siRNA in HL‐1 cells resulted in reduced Igκ staining at cell junctions (Figure 1H) and a significant decrease in Igκ protein levels (Figure 1I), supporting its synthesis by CMs.

To further identify whether Igκ was synthesized by CMs, we separated single CM from µMT and WT mice and detected Igκ transcripts by RT‐PCR using primers for the variable regions and constant regions of the *Igκ* gene. We first identified that rearranged Igκ transcripts were successfully amplified from adult CMs isolated from both WT and µMT mice (Figures 1J and S1F,G). Moreover, all the CM‐derived Igκ transcripts showed classical Vκ‐Jκ rearrangements. Interestingly, each CM showed a unique repertoire, but several dominant pattern of Igκ gene rearrangement frequently observed in different CM (Figure 1K). The dominant Vκ usage in CMs included IgκV17‐121 (28.3%, 95/336), IgκV9‐120 (25%, 84/336), and IgκV14‐100 (15.5%, 52/336), all of which were used at lower frequencies in B cells (Vκ17‐121: 2.1%, Vκ9‐120: 2.5%, and Vκ14‐100: 0.6%) (Figure 1L,M) (data from https://www.10xgenomics.com/). The Igκ transcripts and gene rearrangement were also detected in primary cultured CMs from neonatal mice and HL‐1 cells (Figure S1H‒L).

### 2.2 CM‐Targeted Inhibition of Igκ Results in Arrhythmia and Sudden Death

**FIGURE 2:**
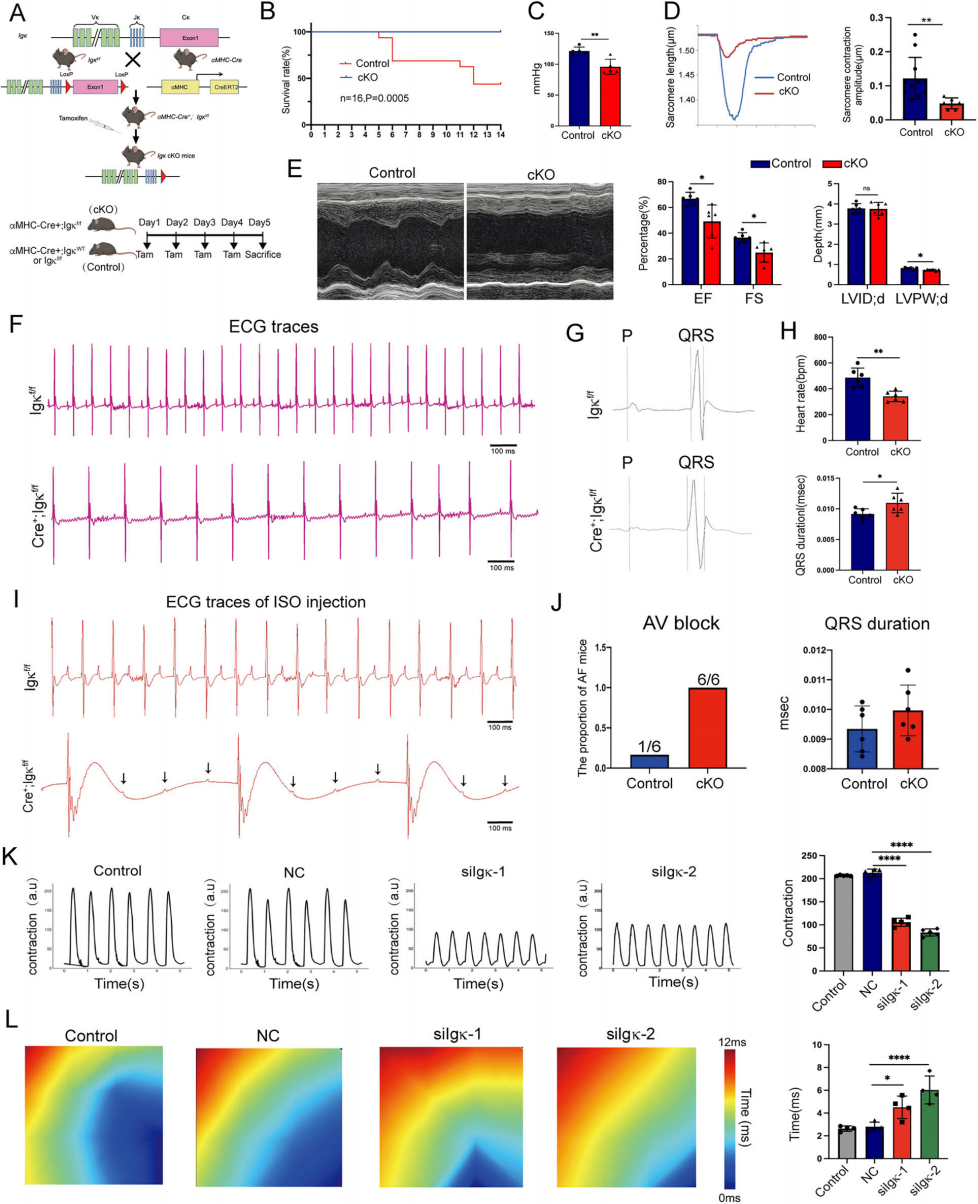
Igκ deficiency led to sudden death, myocardial contraction dysfunction, and conduction defects. (A) Scheme for generation of cardiac conditional Igκ knockout mouse. (B) Survival rate in Igκ^f/f^ mice (control) and Cre+; Igκ^f/f^ mice (cKO) (*n* = 16). Log rank (Mantel‒Cox) test, ^***^ *p* = 0.0005, hazard ratio = 11.57, 95% CI of ratio = 2.936‒45.56. (C) Systolic blood pressure (SBP) in control (*n* = 5) and cKO mice (*n* = 5). Quantitative data are shown as means ± SDs. Statistical analysis was performed with unpaired *t*‐test. ^*^ *p *< 0.05, ^**^ *p* < 0.01, ^***^ *p* < 0.001, ^****^ *p* < 0.0001. (D) Individual traces of sarcomere shortening in isolated ventricular myocytes of control (*n* = 11) and cKO (*n* = 6) mice at 0.5 Hz. Six twitches per myocyte were collected for each sample. Quantitative data are shown as means ± SDs. Statistical analysis was performed with Welch's *t*‐test. ^*^ *p *< 0.05, ^**^ *p* < 0.01, ^***^ *p* < 0.001, ^****^ *p* < 0.0001. (E) Echocardiographic assessment of heart function using M‐mode images of left ventricle and quantification of left ventricular posterior wall (LVPW) thicknesses, left ventricular inner dimension (LVID), left ventricular fractional shortening (FS), and ejection fraction (EF) showed contraction dysfunction in cKO mice (*n* = 6). Quantitative data are shown as means ± SDs. Statistical analysis was performed with multiple *t*‐test. ^*^ *p *< 0.05, ^**^ *p* < 0.01, ^***^ *p* < 0.001, ^****^ *p* < 0.0001. (F) Representative ECG traces for control and cKO mice (*n* = 6). Bar = 100 ms. (G) Representative QRS duration for control and cKO mice (*n* = 6). (H) cKO mice show decreased heart rate and increased QRS duration compared with control mice (*n* = 6). Quantitative data are shown as means ± SDs. Statistical analysis was performed with Welch's *t*‐test. ^*^ *p *< 0.05, ^**^ *p* < 0.01, ^***^ *p* < 0.001, ^****^ *p* < 0.0001. (I) ECG traces from same mice (*n* = 6) observed 10 min post‐isoproterenol (ISO) administration (20 mg/kg, I.P., bar = 100 ms). (J) Incidence of atrioventricular (AV) block and QRS duration in cKO mice (*n* = 6) compared with control mice (*n* = 6) treated with ISO by ECG assay. Quantitative data are shown as means ± SDs. Statistical analysis was performed with Welch's *t*‐test. ^*^ *p *< 0.05, ^**^ *p* < 0.01, ^***^ *p* < 0.001, ^****^ *p* < 0.0001. (K) Video‐based motion analysis showing the beating activity of NC and Igκ knocked down cells (*n* = 5) and the quantified contraction amplitude. Quantitative data are shown as means ± SDs. Statistical analysis was performed with one‐way ANOVA with Dunnett's multiple comparisons. ^*^ *p *< 0.05, ^**^ *p* < 0.01, ^***^ *p* < 0.001, ^****^ *p* < 0.0001. (L) Representative propagation maps of electrical signal and quantified conduction time to compare the conduction function of NC and Igκ knocked down cells (*n* = 4). Quantitative data are shown as means ± SDs. Statistical analysis was performed with one‐way ANOVA with Dunnett's multiple comparisons. ^*^ *p *< 0.05, ^**^ *p* < 0.01, ^***^ *p* < 0.001, ^****^ *p* < 0.0001.

To assess the function of Igκ in CMs, we established a tamoxifen‐induced Igκ knockout (cKO) mouse model by intercrossing Igκ^f/f^ mice with α‐MHC‐Cre mice. Eight to 12‐week‐old cKO mice and their littermates (Igκ^f/f^ and α‐MHC‐Cre negative litermates, control) were administered tamoxifen for 4 consecutive days (Figure 2A). Igκ protein ablation was evaluated using PCR and Western blotting (Figure S2A‒C). Immunofluorescence (IF) staining also showed reduced Igκ level in cKO mice (Figure S2D). Notably, Igκ ablation in CMs led to sudden death in cKO mice as early as day 5, with a survival rate of only 40% after 2 weeks. In contrast, tamoxifen‐treated control mice remained healthy throughout the treatment period (Figure 2B), suggesting that the disease phenotype in cKO mice was not attributable to tamoxifen.

Two days after post‐tamoxifen treatment, we first analyzed cardiac function by measuring the systolic blood pressure. Unexpectedly, cKO mice displayed significantly lower blood pressure (95.8 ± 12.09 mmHg) than control mice (Igκ^f/f^) (121.4 ± 6.427 mmHg, *p* = 0.0056; Figure 2C). Echocardiographic analysis confirmed marked impairment in contractility in cKO mice, with reductions in ejection fraction (EF) (cKO vs. control: 51.87 ± 10.67% vs. 68.26 ± 5.436%) and left ventricular fractional shortening (FS) (cKO vs. control: 24.91 ± 7.497% vs. 36.52 ± 3.880%) (Figure 2E). Additionally, a contractile shortening assay showed that single CMs from cKO mice exhibited a reduced contractile response compared with those from control mice (cKO vs. control: 0.04833 ± 0.01756 µm vs. 0.1189 ± 0.04674 µm) (Figure 2D). These findings highlight the critical role of Igκ in maintaining cardiac contraction function.

We next evaluated the electrophysiological activities of CMs through electrocardiograms, revealing a significant reduction in resting heart rate in cKO mice (cKO vs. control: 341.5 ± 40.45 bpm vs. 486.9 ± 73.42 bpm) and prolongation of QRS duration (cKO vs. control: 0.01096 ± 0.001593 s vs. 0.009176 ± 0.0008318 s) (Figure 2F‒H). The R‒R durations were prolonged in cKO mice (Figure S2E), indicating the presence of a conduction block. To further investigate the implications of reduced Igκ levels and cardiac dysfunction under stress, we injected cKO and control mice with isoproterenol (ISO). As anticipated, ISO injection induced a significantly higher incidence of atrioventricular block in cKO mice (6/6, 100%) compared to control mice (1/6, 17%) (Figure 2I,J). In addition to the increased heart rate induced by ISO, no significant alterations in the QRS interval or RR interval were observed in cKO mice, indicating that Igκ deficiency attenuates the CM responsiveness to ISO stimulation (Figures 2J and S2F).

To further confirm the role of Igκ in human CMs, we knocked down Igκ in human hiPSC‐CMs and analyzed their contractility and conduction ability. The results indicated that knocking down Igκ using two specific siRNAs (Figure S2G) significantly decreased the beating amplitude (212.7 ± 8.050 a.u. for siNC vs. 105.7 ± 8.877 a.u. for siIgκ‐1 and 83.44 ± 7.626 a.u. for siIgκ‐2), suggesting that Igκ deficiency reduces CM contractility (Figure 2K). Furthermore, when plated as a confluent monolayer on a multielectrode array, the conduction time of Igκ‐deficient CMs significantly increased (2.808 ± 0.3928 ms for siNC vs. 4.508 ± 0.9901 ms for siIgκ‐1 and 6.030 ± 1.225 ms for siIgκ‐2) (Figure 2L). Collectively, these in vitro human cellular data demonstrate that Igκ is essential for maintaining CM contractility and electrical conduction, which is consistent with our in vivo murine findings.

### 2.3 Cardiac‐Specific Igκ Knockouts First Resulted in Structural Abnormalities in ICDs

**FIGURE 3:**
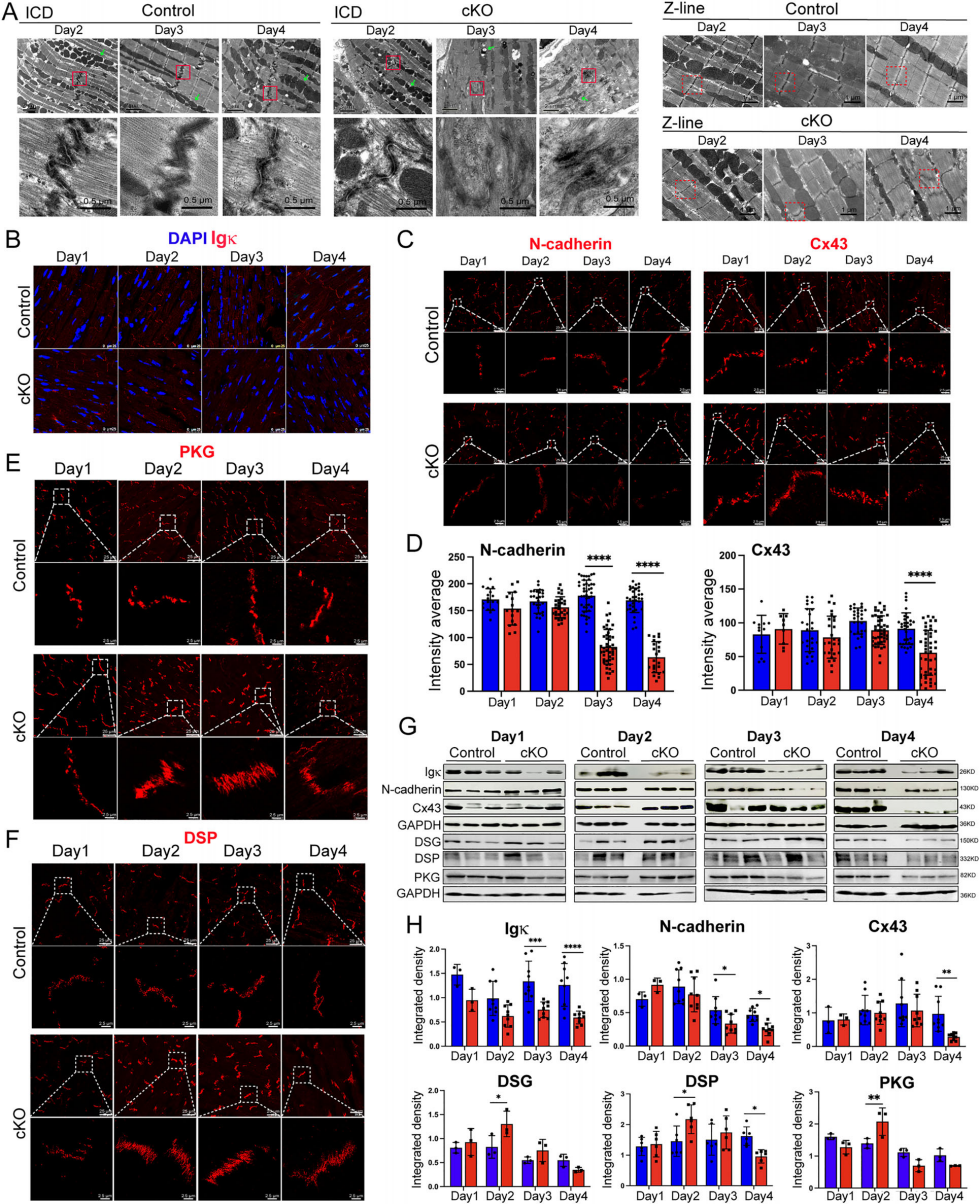
Igκ cKO mice display abnormal structure of cardiomyocyte ultrastructure. (A) The structure of intercalated discs (ICDs) in the heart tissue of control and cKO mice by transmission electron microscopy. cKO mice show severe destruction of ICD structure. The red box indicates the ICDs. The green arrow indicates mitochondria. Scale bar = 2 µm. The ICD zoomed‐in images are shown. Scale bar = 0.5 µm. The structure of Z‐line was showed on the right. Scale bar = 1 µm. The red dotted box represents the Z‐line. (B) Igκ staining (red) in both control and cKO mice for consecutive 4 days after tamoxifen injection. Scale bar = 25 µm. (C) N‐cadherin and connexin 43 (Cx43) staining on the ICDs in both control and cKO mice for consecutive 4 days after tamoxifen injection by stimulated emission depletion microscopy (STED) (red). The scale bar is 25 µm for the representative image and 2.5 µm for the zoomed‐in images. (D) The average intensity of N‐cadherin and Cx43 staining. N‐cadherin: *n* = 17‒45 ICDs of cardiomyocytes from three mice per group. Cx43: *n* = 9‒46 ICDs of cardiomyocytes from three mice per group. Quantitative data are shown as means ± SDs. Statistical analysis was performed with two‐way ANOVA with Sidak's multiple comparisons. ^*^ *p *< 0.05, ^**^ *p* < 0.01, ^***^ *p* < 0.001, ^****^ *p* < 0.0001. (E) Plakoglobin (PKG) staining on the ICDs in both control and cKO mice for consecutive 4 days after tamoxifen injection by STED (red). The scale bar is 25 µm for the representative image and 2.5 µm for the zoomed‐in images. (F) Desmoplakin (DSP) staining on the ICDs in both control and cKO mice for consecutive 4 days after tamoxifen injection by STED (red). The scale bar is 25 µm for the representative image and 2.5 µm for the zoomed‐in images. (G) Expression of Igκ, Cx43, N‐cadherin, desmoglein (DSG), DSP, and PKG on protein level of control (*n* = 3) and cKO (*n* = 3) mice for consecutive 4 days after tamoxifen injection by Western blot. GAPDH was used as an internal control. (H) The integrated density of molecules detected in (G) in control and cKO mice. Quantitative data are shown as means ± SDs. Statistical analysis was performed with two‐way ANOVA with Sidak's multiple comparisons. ^*^ *p *< 0.05, ^**^ *p* < 0.01, ^***^ *p* < 0.001, ^****^ *p* < 0.0001. Three mice were included in each group with three replicates for DSP, Igκ, N‐cadherin, and Cx43 at days 2‒4.

Given the severe conduction dysfunction observed in Igκ‐deficient hearts, we further explored whether Igκ depletion alters the CM ultrastructure. Transmission electron microscopy (TEM) was performed to evaluate the cardiac structure of the cKO and control mice. In control mice, CMs displayed orderly ICDs, well‐organized Z‐lines, and intact mitochondria between adjacent CMs (Figure 3A). In contrast, cKO mice displayed highly convoluted and disorganized ICDs, with widened gaps as early as day 2 after post‐tamoxifen administration (Figures 3A and S3A). By day 3, the Z‐lines began to lose their ordered structure, and the mitochondrial organization became increasingly disrupted (Figure 3A).

To further investigate this, we assessed key ICDs proteins—desmoplakin (DSP), PKG, and desmoglein (DSG) (desmosomal protein), N‐cadherin (an adherens junction [AJ] protein), and connexin 43 (Cx43, a gap junction protein)—using confocal and stimulated emission depletion microscopy (STED). First, IF staining revealed that Igκ‐positive signals on the ICDs were diminished in cKO mice starting on day 2 (Figure 3B), while N‐cadherin levels decreased on day 3 post‐tamoxifen administration, followed by a reduction of Cx43 on day 4 (Figures 3C,D and S3B,C). These temporal alterations indicated that Igκ is critical for maintaining the stable localization of N‐cadherin and Cx43 within ICDs. Consistent with the electron microscopy, STED imaging revealed progressively widened intercellular spaces of desmosomal compartments, including PKG, DSP, and DSG, beginning on day 2 post‐tamoxifen treatment, and was significantly reduced by day 4 (Figures 3E,F and S3D‒F).

Western blotting validated the efficient Igκ knockdown in cKO cardiac tissues. On day 4 post‐tamoxifen treatment, cKO hearts exhibited decreased protein levels of N‐cadherin, Cx43, and desmosomal components (DSP, PKG, and DSG). Collectively, these results demonstrate that Igκ deficiency disrupts ICD integrity by impairing both AJs/gap junctions and desmosomes (Figure 3G,H). In addition, we also found α‐actinin gradually dissolved in the cKO mice heart tissue (Figure S3G), indicating damage to the myocardial cell cytoskeleton and connection function caused by Igκ knockout.

**FIGURE 4:**
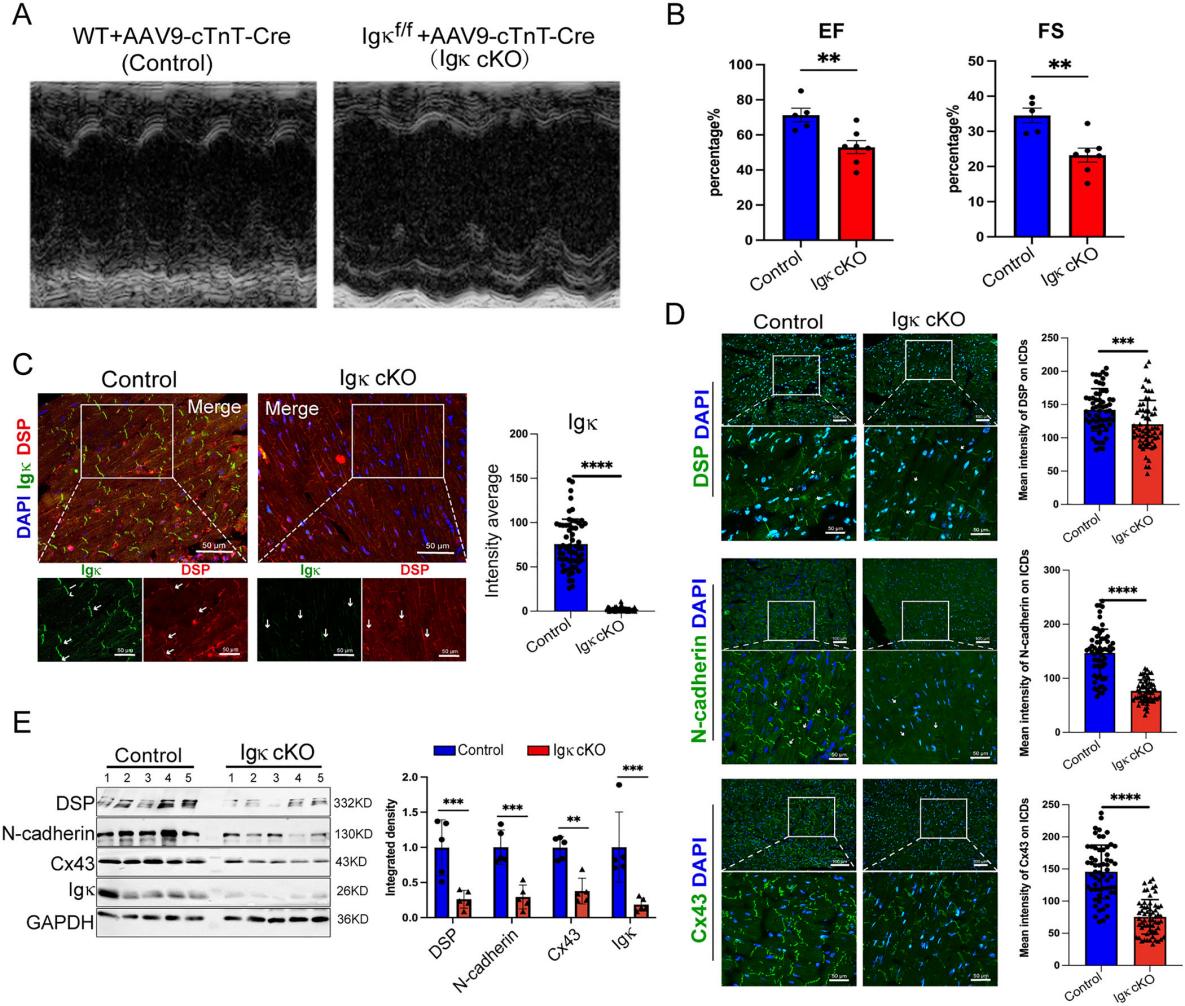
AAV‐induced Igκ deficiency led to myocardial conduction defects and structural abnormalities of ICDs. (A) Echocardiographic assessment of heart function using M‐mode images of left ventricle. (B) Quantification of EF and left ventricular FS showed contraction dysfunction in Igκ cKO mice (*n* = 7). Quantitative data are shown as means ± SDs. Statistical analysis was performed with unpaired *t*‐test. ^*^ *p *< 0.05, ^**^ *p* < 0.01, ^***^ *p* < 0.001, ^****^ *p* < 0.0001. (C) Immunofluorescence staining of Igκ and DSP in WT and Igκ cKO mice (*n* = 3/group). Quantitative analysis showed the average intensity of the Igκ on ICDs were diminished in Igκ cKO mice. Scale bar = 50 µm. Quantitative data are shown as means ± SDs. Statistical analysis was performed with unpaired *t*‐test. ^*^ *p *< 0.05, ^**^ *p* < 0.01, ^***^ *p* < 0.001, ^****^ *p* < 0.0001. (D) The staining of Igκ, DSP, N‐cadherin, and Cx43 in Igκ cKO mice and WT mice. Quantitative analysis showed the average intensity of the Igκ, DSP, N‐cadherin, and Cx43 were all diminished in Igκ cKO mice. Three mice per group were included in the experiment. The scale bar is 100 µm for the representative image and 50 µm for the zoomed‐in images. Quantitative data are shown as means ± SDs. Statistical analysis was performed with unpaired *t*‐test. ^*^ *p *< 0.05, ^**^ *p* < 0.01, ^***^ *p* < 0.001, ^****^ *p* < 0.0001. (E) The protein level of DSP, N‐cadherin, and Cx43 in Igκ cKO mice (*n* = 5) and WT mice (*n* = 5). After normalized to GAPDH loading control, the protein levels of DSP, N‐cadherin, and Cx43 were downregulated in Igκ cKO mice. Quantitative data are shown as means ± SDs. Statistical analysis was performed with two‐way ANOVA with Sidak's multiple comparisons. ^*^ *p *< 0.05, ^**^ *p* < 0.01, ^***^ *p* < 0.001, ^****^ *p* < 0.0001.

To further validate the above findings in an independent knockout model, AAV9‐cTnT‐Cre was administered to Igκ^f/f^ mice via the tail vein to generate an alternative cardiac‐specific Igκ knockout mouse model. Compared to WT mice injected with AAV9‐cTnT‐Cre (WT), Igκ^f/f^ mice injected with AAV9‐cTnT‐Cre (Igκ cKO) displayed reductions in EF and left ventricular FS (Figure 4A,B). IF staining confirmed diminished Igκ signals at DSP‐labeled ICDs, verifying successful Igκ ablation (Figure 4C). Furthermore, both IF staining and Western blot analysis revealed reduced expression of DSP, N‐cadherin, and Cx43 at the ICDs in AAV9‐induced Igκ cKO mice relative to that in WT mice indicating that Igκ deletion disrupts the localization of ICD‐associated proteins (Figure 4D,E). These phenotypic consistencies between tamoxifen‐inducible and AAV9‐mediated Igκ cKO mouse models further confirm that Igκ depletion directly causes ICDs structural disruption and cardiac dysfunction.

**FIGURE 5:**
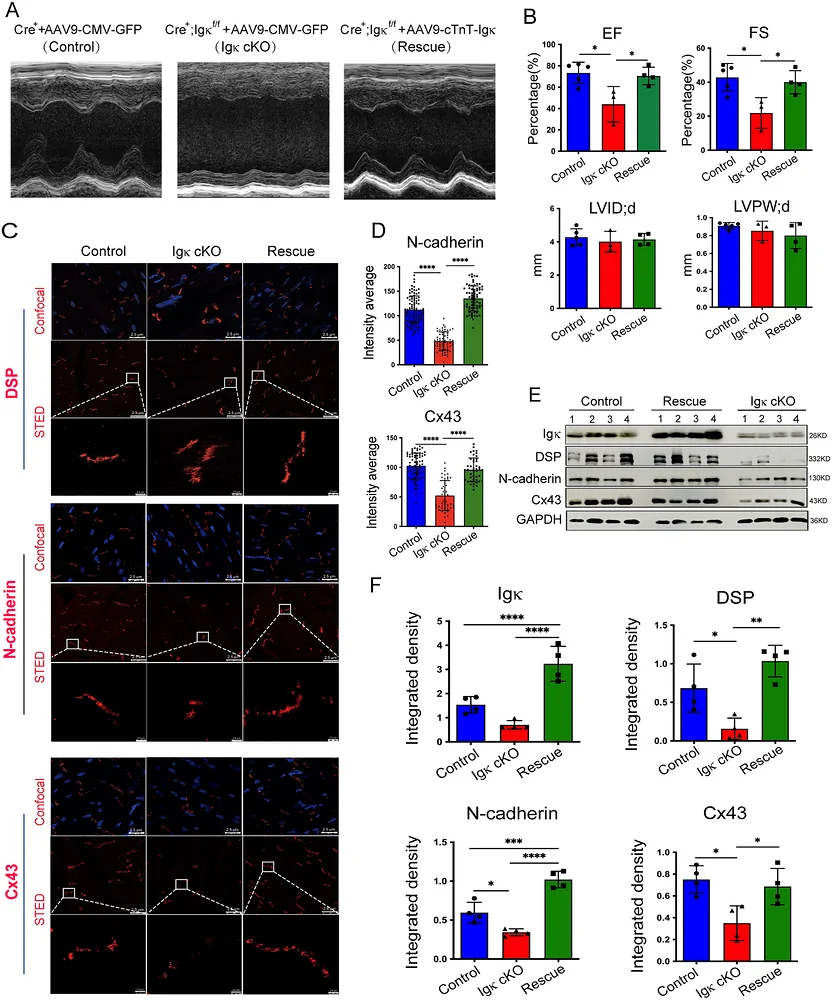
Dysfunction of cardiomyocyte and abnormal structure of ICDs caused by Igκ deficiency can be rescued by overexpression of Igκ using AAV9‐cTnT‐Ig Igκ vector. (A) Echocardiographic assessment of heart function in Igκ cKO mice, control mice, and cKO mice injected with AAV9‐cTnT‐Igκ (rescue group) (*n* = 4/group). (B) Evaluation of cardiac constriction function by EF and FS shows that the cardiac function was improved by overexpressing Igκ in cKO mice. Control: *n* = 5; Igκ cKO: *n* = 3; rescue: *n* = 4. Quantitative data are shown as means ± SDs. Statistical analysis was performed with one‐way ANOVA with Tukey's multiple comparisons. ^*^ *p *< 0.05, ^**^ *p* < 0.01, ^***^ *p* < 0.001, ^****^ *p* < 0.0001. (C) The staining of DSP, N‐cadherin, and Cx43 in cKO mice, control mice, and rescue mice showed exogenous transduction of Igκ improved ICDs structure by confocal and STED. The scale bar is 25 µm for the representative image of confocal and STED. The scale bar of the zoomed‐in images from STED is 2.5 µm. (D) Exogenous transduction of Igκ can rescue the average intensity of the N‐cadherin and Cx43 staining on the ICDs. Quantitative data are shown as means ± SDs. Statistical analysis was performed with one‐way ANOVA with Tukey's multiple comparisons. ^*^ *p *< 0.05, ^**^ *p* < 0.01, ^***^ *p* < 0.001, ^****^ *p* < 0.0001. (E) The protein level of Igκ, DSP, N‐cadherin, and Cx43 in cKO mice, control mice, and rescue mice by Western blot. (F) Quantitative analysis showed the protein levels of DSP, N‐cadherin, and Cx43 were rescued by Igκ transduction in cKO mice (*n* = 4/group). Quantitative data are shown as means ± SDs. Statistical analysis was performed with one‐way ANOVA with Tukey's multiple comparisons. ^*^ *p *< 0.05, ^**^ *p* < 0.01, ^***^ *p* < 0.001, ^****^ *p* < 0.0001.

Moreover, to further confirm Igκ function in ICDs, an AAV9‐cTnT‐Igκ rescue system was constructed. Briefly, Cre^+^;Igκ^f/f^ mice (cKO) were pretreated with AAV9‐cTnT‐Igκ for 4 weeks before tamoxifen‐induced Igκ knockout. Functional assessment showed that exogenous Igκ supplementation (rescue) significantly restored cardiac contractility in cKO mice (rescue vs. cKO, EF: 70.57 ± 8.179% vs. 44.04 ± 16.61%, and FS: 40.01 ± 6.759% vs. 21.89 ± 9.036%) (Figure 5A,B). DSP staining showed that AAV‐mediated Igκ transduction effectively rescued the widened ICDs structure in cKO myocardium (Figure 5C). In addition, exogenous transduction of Igκ stabilized the localization of N‐cadherin and Cx43 on the ICDs (Figures 5C,D and S4). Western blot assays also showed that, the rescue mice reversed the decrease of ICD‐related proteins such as DSP, N‐cadherin, and Cx43 (Figure 5E), confirming that Igκ is required to maintain the normal structure of ICDs by establishing the localization of ICD‐related proteins.

### 2.4 Igκ Knockout in CMs Leads to Plectin Degradation, Resulting in Impaired DSP Anchorage to Desmin and Disruption of the Normal Cytoskeletal Structure

**FIGURE 6:**
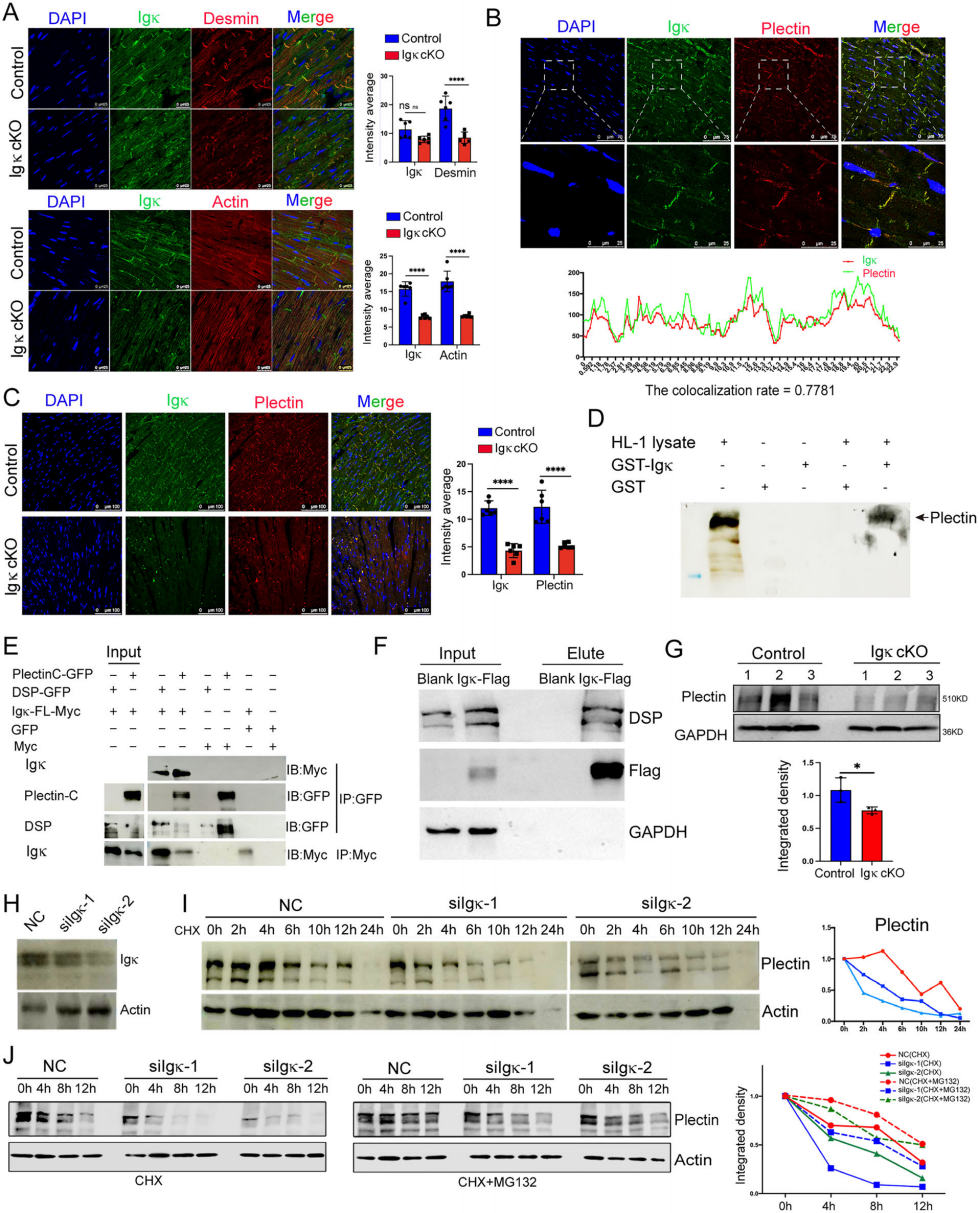
Reduction of Igκ in CM promotes cytoskeletal disorganization by causing degradation of plectin, leading to structural abnormalities in ICDs. (A) Immunofluorescence staining for Igκ, desmin, and actin on the ICDs of Igκ cKO and control mice. Quantitative analysis showed the staining intensity average of Igκ, desmin, and actin were decreased in Igκ cKO mice. Three mice per group with two field were included in the experiment. Scale bar = 25 µm. The scale bar is 75 µm for the representative image and 25 µm for the zoomed‐in images. Quantitative data are shown as means ± SDs. Statistical analysis was performed with two‐way ANOVA with Sidak's multiple comparisons. ^*^ *p *< 0.05, ^**^ *p* < 0.01, ^***^ *p* < 0.001, ^****^ *p* < 0.0001. (B) Colocalization of Igκ and plectin on the ICDs of WT mice by immunofluorescence assay. The colocalization rate is 0.7781. (C) Immunofluorescence staining for Igκ and plectin on the ICDs of Igκ cKO and control mice. Quantitative analysis showed the staining intensity average of Igκ and plectin was decreased in Igκ cKO. Scale bar = 100 µm. Three mice per group with two field were included in the experiment. Quantitative data are shown as means ± SDs. Statistical analysis was performed with two‐way ANOVA with Sidak's multiple comparisons. ^*^ *p *< 0.05, ^**^ *p* < 0.01, ^***^ *p* < 0.001, ^****^ *p* < 0.0001. (D) Results of GST pull‐down experiment by using GST‐Igκ/GST and HL‐1 cell lysate. Western blot was used to identify plectin and GST. (E) Plectin‐C‐GFP/DSP‐GFP and Igκ‐Myc were co‐transfected into 293T cells and total cell lysate was extracted and subjected to IP assay using GFP antibody. Western blot was used to detect Igκ, plectin‐C, and DSP by using anti‐Myc and anti‐GFP antibody. (F) Flag‐tagged Igκ was overexpressed in AC16 cells by lentivirus vector and immunoprecipitated with anti‐Flag antibody. Input and eluted proteins were analyzed by Western blot for DSP. (G) Expression of plectin in the heart tissue of Igκ cKO and control mice by Western blot assay. Quantitative data are shown as means ± SDs. Statistical analysis was performed with unpaired *t*‐test. ^*^ *p *< 0.05, ^**^ *p* < 0.01, ^***^ *p* < 0.001, ^****^ *p* < 0.0001. (H) The expression of Igκ was decreased in HL‐1 cells by using two specific siRNA for Igκ. (I) Igκ knocked down resulted in a rapid degradation of plectin protein in HL‐1 cells. Cells were co‐treated with cycloheximide (CHX), and harvested at 0, 2, 4, 6, 10, 12, and 24 h after the treatments for Western blot analysis. Line chart of plectin on protein level that are normalized to actin loading control. (J) MG132 was used to inhibit the degradation of plectin via proteasome in both Igκ knocked down cells and control cells treated with CHX. Line chart of plectin on protein level that are normalized to actin loading control.

ICDs and Z‐lines are essential mechanical structures in CMs, whose structural integrity of which relies on coordinated crosstalk between intermediate filaments and microfilaments (especially actin filaments). Desmosomes physically connect adjacent CMs by anchoring intermediate filaments, whereas cadherin complexes mechanically link cell junctions to the actin cytoskeleton to strengthen intercellular adhesions. The dissociation of actin or desmin (an intermediate filament protein) from ICDs triggers the disassembly of desmosomes and AJ components [24, 25]. Accordingly, we next investigated whether Igκ depletion disrupts cytoskeletal organization. IF results showed that both desmin and F‐actin staining on ICDs were significantly diminished in the heart tissues of Igκ cKO mice compared to control mice (Figure 6A). Similarly, the filamentous structures of desmin and F‐actin in HL‐1 cells were disrupted when Igκ was knocked down by specific siRNA (Figure S5A), suggesting that a lack of Igκ also led to abnormal assemble of F‐actin and desmin.

Next, we explored the molecular mechanism by which Igκ regulates intermediate filament and microfilament assembly in ICDs. In our previous study, we found that epithelial cancer cell‐derived Igκ interacts with plectin, which can bind to and promote the assemble of both intermediate filaments and actin to maintain the integrity of the cytoskeletal network. Loss of Igκ induces plectin degradation and the subsequent cytoskeletal disassembly (unpublished data). In addition, in CM, plectin has been regarded as to bind with DSP and connect DSP to desmin [26, 27, 28, 29]. Thus, we hypothesized that Igκ may bind with plectin, not only promote the assemble of desmin and actin, but also to connect desmin with DSP to maintain normal ICDs structure. First, we analyzed whether Igκ interacted with plectin. The results showed that Igκ and plectin were colocalized on the ICDs of CMs in WT mice, as well as on the cell junctions of HL‐1 cells (Figures 6B and S5B). Moreover, Igκ knockdown can significantly lead to decreased plectin on the ICDs of mice, and HL‐1 cell junctions (Figures 6C and S5B). A GST pull‐down assay was further performed to verify the direct interaction between Igκ and plectin. Plectin was significantly enriched in the GST‐Igκ group relative to that in the GST control group, confirming their interaction (Figure 6D). For further investigation, we established an expression vector containing the C‐terminus of plectin (the whole molecular weight of plectin is over 500 kDa, which is difficult to be expressed exogenously), which can bind with the intermediate filaments (Figure S5C). The plectin‐C vector was co‐transfected with distinct Igκ constructs (variable region, constant region, and full‐length Igκ) into 293T cells. Subsequent co‐IP assays validated the binding between plectin‐C and Igκ, and this interaction was predominantly dependent on the Igκ variable region (Figures 6E and S5D). We also found that Igκ can bind with DSP (Figure 6E,F), indicating that Igκ can bind DSP on the ICDs with plectin, which can bind with desmin and maintain the integrity of the ICDs structure.

We further investigated the effect of Igκ knockdown on plectin. We first analyzed whether Igκ knockdown reduced plectin transcription in CM compared with that in control cells and found that the mRNA level of plectin showed no difference (data not shown). Subsequently, we further analyzed whether Igκ knockout reduced plectin on protein levels in Igκ cKO mice compared with control mice and found that plectin was reduced in Igκ cKO mice (Figure 6G). To address whether lack of Igκ could decrease the protein degradation of plectin, cycloheximide (CHX) was used to inhibit protein synthesis. The results showed that a lack of Igκ could significantly accelerate the decrease of plectin (Figure 6H,I), and MG132 (a proteasome inhibitor) could obviously delay the rapidly degradation of plectin (Figure 6J). In addition, it has been reported that CHX distortion may affect mRNA and translational efficiency measurements. Therefore, we also analyzed the mRNA level of plectin and found that although CHX inhibited plectin mRNA production but there was no significant decrease in Igκ knockdown cells compared with control cells (Figure S5E,F).

## 3 Discussion

We report for the first time that the Igκ light chain, traditionally considered as an immune molecule, is widely expressed in both murine and human CMs. Our findings show that Igκ is predominantly localized on the ICDs of CMs and plays a key role in maintaining CM structural integrity, myocardial contraction, and conduction. These results challenge classical immunological theories that have long posited that Igs are exclusively produced by B cells. However, phenomena such as the rapid appearance of free Ig light chains or IgE in the blood following acute myocardial infarction, and the presence of autoantibodies [30, 31, 32, 33] against cardiac structural proteins in myocardial injury diseases, cannot be fully explained by the classical immunological theory that Igs can only be produced by B cells. Our findings suggest that these “autoantibodies” can also be produced by injured CMs and exert biological roles beyond classic immune responses.

Our previous work revealed that CMs can widely express different classes of Ig, including IgM and IgG [23], as well as the Igκ light chain. However, their physiological function remains unclear. In this study, we focused on the Igκ light chain to investigate its physiological function in CMs. We first provided evidence of Igκ expression at the protein and transcriptional levels in both murine and human CMs. Our results confirmed that Igκ is expressed on the cell membrane and sarcomere, particularly on ICDs, where the signal is the strongest.

Next, we explored the characteristics of Igκ variable region usage in CMs. Single‐cell sequencing revealed that Igκ in CMs of adult mice exhibits a conservative VκJκ usage, predominantly expressing IgκV17‐121 (28.3%, 95/336), IgκV9‐120 (25%, 84/336), and IgκV14‐100 (15.5%, 52/336). It is worth noting that the conservative characteristics of CM‐derived Igκ were similar to those of Igκ in hepatocytes and other non‐B cells, especially IgκV9‐120, which has been found to be highly frequently expressed in other non‐B‐cell lineages [8]. However, the expression frequency of the above VκJκ usages in B cells was less than 3%. This limited diversity suggests that the Igκ light chain in CMs may not exert immune functions. Instead, it appears to play a physiological role in maintaining normal heart functions.

To further investigate the physiological function of Igκ, we generated conditional Igκ knockout (cKO) mice with tamoxifen inducible deletion of Igκ in CMs. Tamoxifen‐induced knockout of Igκ resulted in significant decreases in blood pressure, cardiac EF, and heart rate, beginning as early as 2 days post‐induction. The knockout mice also exhibited more frequent atrioventricular blocks in response to ISO than the controls. Notably, approximately 60% of the Igκ knockout mice died within 2 weeks, underscoring the critical role of Igκ in maintaining cardiac function. Similarly, Igκ knockout in human iPSC‐derived CMs led to impaired contractility and conduction, further confirming its essential role in CM function.

Next, we investigated the mechanisms underlying the cardiac dysfunction in the Igκ‐deficient mice. TEM revealed structural disorganization of the ICDs, with a loss of N‐cadherin and Cx43 localization on the ICDs. This disruption was associated with impaired myocardial contraction and conduction, suggesting that Igκ plays a vital role in stabilizing ICDs structure. To further validate this hypothesis, we used AAV9‐cTnT‐Igκ to overexpress Igκ in CMs. This overexpression prevented the structural and functional disruptions observed in Igκ knockout mice, confirming that these changes were indeed due to Igκ deficiency.

ICDs are composed of three major complexes, namely, desmosomes, AJs, and GJs. Desmosomes and AJs provide mechanical attachment between two CMs by anchoring the intermediate filaments and actin, thereby enhancing the stability of the ICDs. GJs metabolically and electrotonically connect the cytoplasm of adjacent CMs metabolically and electrotonically to enable the propagation of electrical stimuli throughout heart muscle cells. These three complexes work together to incorporate the mechanical and electrical functions of the ICDs [34, 35, 36, 37]. Notably, components of desmosomes and AJs are intimately associated within the area composition and work together to facilitate mechanical coupling. In the area composita, desmosomes are considered to be associated with the intermediate filament desmin through binding to DSP, and AJs are considered to be associated with actin through binding α‐catenin, β‐catenin, and vinculin [24, 25]. Cytoskeletal abnormalities can cause DSP and AJs to be unable to be properly located on the ICDs. Therefore, we further determined the intracellular localization of both F‐actin and desmin and found that Igκ knockout also resulted in the disassembly of both F‐actin and desmin. Therefore, we propose the scientific question of how Igκ simultaneously affects the assembly and positioning of actin and intermediate filaments. Our study revealed that Igκ in CMs can interacts with plectin. Plectin is a large cytolinker protein whose main function is to interact and promote the assembly of actin and intermediate filaments [38, 39, 40, 41]. It has been reported that plectin is localized on ICDs and connects the intermediate filaments/desmin to desmosomes by interacting with DSP [26, 27, 28]. Our results showed that Igκ interacted with plectin depending on its variable region. Additionally, Igκ knockout resulted in plectin degradation. Loss of plectin leads to the disassembly of actin and desmin, resulting in disconnection between the desmosomes and AJs. This leads to abnormal electrical coupling of the CMs and a reduced conduction velocity. This disruption in the cytoskeleton likely underpins the high mortality observed in Igκ‐deficient mice.

Critically, CM‐specific deletion of Igκ triggers structural disorganization of ICDs, which further causes reduced myocardial contractility and conduction block, ultimately predisposing mice to sudden cardiac death. This pathological phenotype closely recapitulates the clinical and pathological manifestations of arrhythmogenic cardiomyopathy (ACM) [42]. Studies have shown that biopsy tissues from ACM patients with ACM exhibit no obvious abnormalities under light microscopy; however, electron microscopy reveals ICDs structural defects, including aberrant desmosomal localization and widening of intercellular spaces [43]. Currently, desmosomal gene mutations that trigger a cascade of ICDs remodeling are recognized as the core pathogenic mechanism of ACM. Nevertheless, the full spectrum of gene mutations accountable for ACM has not yet been identified. Our study provides a significant extension to the current paradigm: deficiency of the non‑desmosomal gene Igκ also results in the disintegration of ICDs. This finding offers a new perspective for understanding how non‑desmosomal gene mutations contribute to ACM progression. Moreover, growing evidence suggests that enhancing intercellular cohesion to stabilize the ICDs represents a promising therapeutic strategy for ACM [44]. Our work demonstrates that Igκ maintains normal ICDs structure by connectin desmin and desmosomal complexes. Accordingly, it is worth exploring whether Igκ transduction in ACM CMs could consolidate ICD structure and confer therapeutic benefits.

This study had several limitations in this study. Although we verified that Igκ binds to plectin and prevents its degradation via the proteasome pathway, the exact binding sites between Igκ and plectin, as well as the detailed molecular mechanism by which Igκ inhibits plectin degradation, remains unclear. In addition, this study only explored the physiological functions of CM‐derived Igκ, while its pathological roles in cardiac diseases have not been elucidated, limiting further explanation of its clinical translational value.

## 4 Conclusion

In summary, our study has made a significant breakthrough by providing evidence that Igκ, which is traditionally considered an immune molecule, is expressed in CMs and plays a critical structural role. Specifically, Igκ interacts with plectin and forms a complex with DSP to stabilize ICDs. This interaction is crucial for maintaining the integrity of the desmosomal complexes, which in turn supports proper myocardial contraction and conduction. These findings not only expand our understanding of cardiac physiology but also introduce Igκ as a potential therapeutic target. By enhancing the stability of ICDs, interventions aimed at Igκ may offer a novel approach for treating heart‐related diseases.

## 5 Material and Method

### 5.1 Human Heart Specimen

Human heart autopsy specimens were obtained from the volunteer donors. All heart tissue acquisitions were conducted using protocols approved by the Institutional Review Board of Fuwai Hospital, Chinese Academy of Medical Sciences (approval no. 2021‐1465). We have complied with all relevant ethical regulations and obtained the written informed consent was obtained from all participating patients.

### 5.2 Animal Studies

Adult Balb/c mice and C57BL/6 mice (8 weeks old) were obtained from Vital River (Charles River Laboratories) and µMT mice (Balb/c background) were a gift from Professor Zhihai Qin (Institute of Biophysics, Chinese Academy of Sciences). The mice were perfused with PBS for 5 min, and the heart was removed. Animal housing conditions and all experimental procedures were conducted in accordance with the institutional guidelines provided by the Institutional Animal Care and Use Committee of China. The animal studies were approved by the Peking University Biomedical Ethics Committee (LA2022295).

### 5.3 Generation of Conditional Cardiac‐Specific Igκ‐Knockout Mice

To generate inducible cardiac‐specific Igκ cKO mice, transgenic mice expressing a tamoxifen‐inducible Cre recombinase protein under the control of the α‐myosin heavy chain promoter, αMHC/MerCreMer mice (MCM) were intercrossed with Igκ^flox/flox^ mice (obtained from Shanghai Biomodel Organism Science & Technology Development Co., Ltd) in a C57BL/6 genetic background. To induce Cre recombination, 8‒12‐week‐old male αMHC/MerCreMer; Igκ^flox/flox^ (Cre^+^; Igκ^f/f^) mice were treated with 80 mg/kg of tamoxifen (Sigma) via intraperitoneal injection once a day for 4 consecutive days. αMHC‐Cre^−^; Igκ^f/f^ mice and αMHC‐Cre^+^ mice with tamoxifen age‐ and sex‐matched littermates were utilized as control mice. Animal housing conditions and all experimental procedures were conducted in accordance with the Institutional Animal Care and Use Committee of China.

### 5.4 HL‐1 Cell Culture

HL‐1, an adult mouse atrial myocardial cell line, was a gift from Dr. W.C. Claycomb (Louisiana State University, New Orleans, LA) [45]. HL‐1 cells were cultured in Claycomb medium (Sigma‒Aldrich) supplemented with 10% FBS (HyClone), 100 U/mL penicillin, 100 µg/mL streptomycin, 2 mM L‐glutamine, and 0.1 mM norepinephrine (Sigma‒Aldrich) at 37°C under 5% CO_2_ in 0.02% gelatin‐coated flasks containing 0.5% fibronectin (Sigma‒Aldrich).

### 5.5 Isolation of Adult Mouse Ventricular Myocytes and Single CM Dissociation

Ventricular myocytes were isolated from the Langendorff‐perfused hearts of adult C57BL/6 mice. Briefly, the mice were anesthetized with pentobarbital and the heart was removed and mounted on the Langendorff apparatus and perfused with Ca^2+^‐free Tyrode's solution to eliminate the blood. Then, the heart was perfused with Tyrode's solution containing 0.67 mg/mL of collagenase type II (Worthington) for 30 min for digestion. When the heart became flaccid, remove the ventricle and cut into small pieces and then pelleted by centrifugation at 500 rpm for 1 min at room temperature and resuspended in Tyrode's solution. The calcium concentration was then gradually increased to 500 M over 80 min. The supernatant was discarded, and the pellet was resuspended in PBS containing 1 mg/mL BSA. A single CM suspension was gently sucked into the micro‐capillary with a mouth pipette [46]. To ensure that only single cells were collected, the solution was visually inspected under a microscope and was discarded if multiple cells were observed. The volume of the liquid was kept below 0.5 µL. Cells were then transferred to a 0.2 mL thin‐wall PCR tube containing lysate buffer.

### 5.6 RNA Extraction and cDNA Synthesis

Myocardial single‐cell RNA extraction and cDNA synthesis were performed out according to Tang's methods as previously described [47]. In briefly, myocardial single cells were picked using a capillary pipette and reverse transcription was then performed directly on the whole cell lysate. After this, the free primers were removed using ExoSAP‐IT and a poly(A) tail was added to the 3′ end of the first‐strand cDNA by Terminal Deoxynucleotidyl Transferase. Then, the single‐cell cDNAs were amplified following PCR program: 95°C for 3 min, then 27 cycles of 95°C for 30 s, 67°C for 1 min, and 72°C for 6 min (+6 s each cycle). After this step, all cDNAs were amplified. The single‐cell cDNA PCR product can be saved at −80°C for 2 months.

Total RNA was extracted from frozen heart tissue and HL‐1 cells by using Trizol Reagent (Invitrogen). Reverse transcription (RT) was performed using the First Stand cDNA Synthesis Kit (Thermo Scientific) according to the manufacturer's protocol.

### 5.7 Construction of Adenovirus

The AAV serotype 9 vector‐cTnT‐Cre (AAV9‐cTnT‐Cre) was produced by WZ Biosciences. 4 × 10^11^ vg/g AAV9‐cTnT‐Cre were injected intravenously into 8‐week‐old Igκ^f/f^ mice and WT mice. Four weeks after injection, the mice were harvested for relevant detection.

The full‐length cDNA of mouse IgκV17‐121/J2/C was inserted into the AAV serotype 9 vector‐cTnT (AAV9‐cTnT) to construct the AAV9‐cTnT‐Igκ virus (produced by Likeli, Beijing, China). 5 × 10^11^ vg/g of AAV9‐cTnT‐Igκ or AAV9‐CMV‐GFP was injected intravenously into 10‐week‐old cardiac Igκ knockout mice (αMHC‐Cre^+^; Igκ^f/f^), whereas the same volume of AAV9‐CMV‐GFP was injected intravenously into αMHC‐Cre^+^ mice. Four weeks after injection, tamoxifen was injected to induce Igκ gene knockdown. Western blot of myocardial tissues was performed to confirm the re‐expression efficiency.

### 5.8 Statistical Analysis

All data are presented as mean ± SD and were analyzed using Prism GraphPad software v8.0. Statistical analysis of the two experimental groups was performed using unpaired Student's *t*‐test. Comparisons of more than two groups were performed using one‐way analysis of variance (ANOVA), and comparisons of two groups with different time points were performed using two‐way ANOVA. The effect size was calculated using SPSS, and the power was calculated using the OpenEpi and PASS software. Hierarchical analysis was performed using Stata SE software. Statistical significance was set at *p* < 0.05 (^*^ *p* < 0.05, ^**^ *p* < 0.01, ^***^ *p* < 0.001, ^****^ *p* < 0.0001).

Detailed materials and methods can be found in the Supporting Information.

## Author Contributions

Z.Z. and Q.X.Y. conceived the study and designed the work plan. Z.Z., S.Y.X., C.Y., Z.W.J., and S.Z. performed experimental work. Development and contribution of reagents were conducted by L.F., L.Q.Y., and Z.M. Statistical analysis was performed by Z.Z., S.Y.X., and W.W.X. Manuscript writing was done by Z.Z., S.Y.X., Q.X.Y., S.J.P., and L.F. All the authors have read and approved the final manuscript .

## Funding

This work was supported by research grants to X. Qiu from the National Science Foundation of China (82030044 and 82341227), grants to Feng Lan from Shenzhen Medical Research Fund (B2302048) and National High Level Hospital Clinical Research Funding (2025‐GSP‐GG‐26), and grants to Zhu Zhu from the National Science Foundation of China (82202023) and grants to Jiangping Song from the National Science Foundation for Distinguished Young Scholars of China (82125004).

## Ethics Statement

Human heart autopsy specimens were obtained from the volunteers. All heart tissue acquisitions were conducted using protocols approved by the Institutional Review Board of Fuwai Hospital, Chinese Academy of Medical Sciences (approval no. 2021‐1465). We complied with all relevant ethical regulations and obtained written informed consent from all participating patients. Animal housing conditions and all experimental procedures were conducted in accordance with the institutional guidelines provided by the Institutional Animal Care and Use Committee of China. The animal studies were approved by the Peking University Biomedical Ethics Committee (LA2022295).

## Conflicts of Interest

The authors declare no conflicts of interest.

## Supporting information




**Supporting File 1**: mco270940‐sup‐0001‐SuppMat.docx

**Supporting File 2**: mco270940‐sup‐0002‐SuppMat.xlsx

**Supporting File 3**: mco270940‐sup‐0003‐SuppMat.xlsx

## Data Availability

Detailed materials and methods can be found in the Supporting Information. The raw sequence data reported in this paper have been deposited in the Genome Sequence Archive (GSA) at CNCB/BIG under accession CRA045100 via https://ngdc.cncb.ac.cn/gsa/s/TndIhM0E. Additional data that supporting the findings of this study are available from the corresponding author upon reasonable request.
