# Supplementary material for: Immunoglobulin Kappa Light Chain Produced by Cardiomyocytes and Participates in Maintaining Intercalated Disc Integrity
Source: MedComm (2020). 2026 Sep 9;7(9):e70940. doi: 10.1002/mco2.70940 (PMC13555800; doi:10.1002/mco2.70940)
Supplement: Supplementary file 1 [file MCO2-7-e70940-s003.docx]

**Supporting information for**

**Immunoglobulin kappa light chain produced by cardiomyocytes and participates in maintaining intercalated disc integrity**

**Running Title:** Zhu et al; Immunoglobulin kappa maintains ICDs integrity

Zhu Zhu, PhD^1,2,3^; et al

^1.^ NHC Key Laboratory of Medical Immunology, Peking University, Beijing, China.

^2.^Key Laboratory of Molecular Immunology, Chinese Academy of Medical Sciences, Beijing, China.

^3.^Department of Immunology, School of Basic Medical Sciences, Peking University, Beijing, China.

**Material and method**

**1. Human heart specimen**

Human heart autopsy specimens were obtained from the volunteer donors. All heart tissue acquisitions were conducted using protocols approved by the Institutional Review Board of Fuwai Hospital, Chinese Academy of Medical Sciences (Approval No. 2021-1465). We have complied with all relevant ethical regulations and obtained the written informed consent was obtained from all participating patients.

**2. Animal Studies**

Adult Balb/c mice and C57BL/6 mice (8 weeks old) were obtained from Vital River (Charles River Laboratories) and μMT mice (Balb/c background) were a gift from Professor Zhihai Qin (Institute of Biophysics, Chinese Academy of Sciences). The mice were perfused with PBS for 5 minutes, and the heart was removed. Animal housing conditions and all experimental procedures were conducted in accordance with the institutional guidelines provided by the Institutional Animal Care and Use Committee of China. The animal studies were approved by the Peking University Biomedical Ethics Committee (LA2022295).

**3. HL-1 Cell culture**

HL-1, an adult mouse atrial myocardial cell line, was a gift from Dr. W.C.Claycomb (Louisiana State University, New Orleans, LA)[1]. HL-1 cells were cultured in Claycomb medium (Sigma-Aldrich) supplemented with 10% FBS (HyClone), 100 U/ml penicillin, 100 μg/ml streptomycin, 2 mM L-glutamine, and 0.1 mM norepinephrine (Sigma-Aldrich) at 37°C under 5% CO_2_ in 0.02% gelatin coated flasks containing 0.5% fibronectin (Sigma-Aldrich).

**4. MEA Recordings of hiPSC-CMs**

The hiPSC-CMs were obtained by volunteer donors. The hiPSC-derived cardiomyocytes were digested in CardioEasy CM dissociation buffer, after which 5 × 10^4^ cells were plated on a microelectrode array (MEA) pre-coated with 5% Matrigel (Cellapy). The next day, 300-μl medium was added to each well. After the cardiomyocytes resumed spontaneous beating, the experimental data were recorded on a Maestro EDGE (Axion Biosystems, Inc., Atlanta,CA, USA) according to the MEA manual. AxIS Navigator and Origin software were used to analyze the data.

**5. Quantification of the contraction of hiPSC-CMs**

Purified cardiomyocytes were seeded into the plate, and cell contractility was recorded when spontaneous beating was restored. We used the software tool "MUSCLEMOTION,” developed by Christine et al[2] , t to record a video of the beating of cardiomyocytes to measure the contractile force of cardiomyocytes in vitro. A mathematical algorithm model was used to analyze the changes in pixels at a certain point during cardiomyocyte beating and then convert them into related parameters of myocardial contraction. The data obtained were analyzed using ImageJ and Origin software.

**6. Isolation and culture of neonatal mouse ventricular myocytes**

Ventricular cardiomyocytes were isolated from the left ventricles of neonatal mice using enzymatic digestions with 0.08% trypsin (Gibco BRL) and 0.1% collagenase (Sigma-Aldrich) for 8 min at 37 °C. Isolated cardiomyocytes were resuspended in Dulbecco’s modified Eagle’s medium (DMEM) containing 10% fetal bovine serum (FBS) and 1% penicillin–streptomycin and then plated at a density of 6 × 10^5^/dish and cultured for 1.5 h. The supernatant was subsequently collected and centrifuged at 500g for 10 min and resuspended in 5 ml culture medium containing 100 mol/L 5’BrdU (Sigma-Aldrich) and plated in 35 mm culture dishes for 48h.

**7. Isolation of adult mouse ventricular myocytes and single cardiomyocyte dissociation.**

Ventricular myocytes were isolated from the Langendorff-perfused hearts of adult C57BL/6 mice. Briefly, the mice were anaesthetized with pentobarbital and the heart was removed and mounted on the Langendorff apparatus and perfused with Ca^2+^ -free Tyrode’s solution to eliminate the blood. Then the heart was perfused with Tyrode’s solution containing 0.67mg/ml of collagenase type II (Worthington) for 30 minutes for digestion. When the heart became flaccid, remove the ventricle and cut into small pieces and then pelleted by centrifugation at 500rpm for 1 min at room temperature and resuspended in Tyrode’s solution. The calcium concentration was then gradually increased to 500M over 80 min. The supernatant was discarded, and the pellet was resuspended in PBS containing 1mg/ml BSA. A single cardiomyocyte suspension was gently sucked into the micro-capillary with a mouth pipette[3]. To ensure that only single cells were collected, the solution was visually inspected under a microscope and was discarded if multiple cells were observed. The volume of the liquid was kept below 0.5 μl. Cells were then transferred to a 0.2 ml thin-wall PCR tube containing lysate buffer.

**8. RNA extraction and cDNA synthesis**

Myocardial single cell RNA extraction and cDNA synthesis were performed out according to Tang’s methods as previously described [4]. In briefly, myocardial single cells were picked using a capillary pipette and reverse transcription was then performed directly on the whole cell lysate. After this, the free primers were removed using ExoSAP-IT and a poly(A) tail was added to the 3’ end of the first-strand cDNA by Terminal Deoxynucleotidyl Transferase. Then the single cell cDNAs were amplified following PCR program: 95 °C for 3 min, then 27 cycles of 95 °C for 30 sec, 67 °C for 1 min, and 72 °C for 6 min (+ 6 sec each cycle). After this step, all cDNAs were amplified. The single cell cDNA PCR product can be saved at −80 °C for 2 months.

Total RNA was extracted from frozen heart tissue and HL-1 cells by using Trizol Reagent (Invitrogen). Reverse transcription (RT) was performed using the First Stand cDNA Synthesis Kit (Thermo Scientific) according to the manufacturer’s protocol.

**9. PCR amplification and DNA sequencing**

After the cDNA was obtained, specific primers were used to amplify the Igκ, CD20, Myh6, Myh7, cTnT, cTnI and GAPDH. The sequences of all the PCR primers are listed in Table S1. Touchdown PCR was performed for amplification. The PCR products were cloned into a pGEM-T Easy Vector (Promega). These clones were analyzed by the Sanger sequencing method using an ABI 3100 Genetic Analyzer (Applied Biosystems). The sequences of VκJκ were compared with those in the BLAST and Immunogenetics databases to identify the best matches for germline gene segments and VJ junctions. All VκJκ sequences are shown in Data set EV1.

For the next-generation sequencing, primers with different barcodes (Table S2) in the second round of PCR were used to distinguish different cells and the DNA concentration of eluted PCR products was measured and 146 ng of DNA was pooled for sequencing. The following sequencing using the a 2 × 250 bp Illumina MiSeq platform were performed by Novogene Corporation (Beijing, China). Single cell from WT mice (n=17) and μMT mice (n=3) were analysed. The usage of Vκ was shown in dataset EV 2.

**10. RNA interference**

The sequence for mouse Igκ siRNA was as follows: siRNA-1: GAAGATTGATGGCAGTGAA. siRNA-2: GAACGACATAACAGCTATA. The sequence for human Igκ siRNA was as follows: siRNA-1: CCUCCUAGGCUGCUCAUUUA. siRNA-2: CCUGGGACCAAAGUGGAAAT. These were used to transfect HL-1 cells or iPSC-CMs using the Lipofectamine 3000 kit (Invitrogen) according to manufacturer’s instructions; a mock group was transfected with control siRNA. Cells were harvested 24 h and 48 h after transfection for Igκ mRNA and protein detection.

**11. Immunohistochemistry (IHC)**

For the detection of Igκ, the tissue sections were deparafinized and rehydrated through graded ethanol washes. Antigen retrieval was performed in 10 mM citrate buffer (pH 6.0) and boiled for 2 min. The slides were incubated with 3% hydrogen peroxide for 10 min to eliminate the endogenous peroxidase. The slides were then blocked with 10% normal goat serum for 30 min at RT. After removing excess blocking buffer, indirect immunohistochemical staining was performed with anti-Igκ primary antibody at 4°C overnight, washed thoroughly, and then incubated with anti-rabbit/mouse IgG horseradish peroxidase (Dako) at RT for 30 min. The slides were washed again, and the bound antibodies were detected using diaminobenzidine.

**12. Immunofluorescence (IF)**

Frozen sections of heart tissues, primary cultured cardiomyocytes, HL-1 cells and hiPS-CMs were fixed with acetone or paraformaldehyde (for F-actin staining). For F-actin staining, cells were permeabilized with 0.5% Triton X-100 for 10 min at room temperature. Then blocked with 5% FBS at room temperature for 30 minutes. The primary antibodies were added at 4°C overnight. The cells were then washed with PBS and incubated for 1 h with the respective secondary antibodies, after which the cells were stained with Hoechst (1:10000; Sigma) for 5min at room temperature. The antibodies and dilution ratios are shown in Tables S3 and Table S4.

**13. Histological analysis**

The results of sections staining were quantified using Fiji-ImageJ software. The mean intensity of Igκ, Plakoglobin, Cx43, DSP, N-cadherin on ICDs was determined using the freehand line in the ImageJ toolbar. Ten random fields of a human sample were selected for fluorescence intensity and statistical analyses. Twenty ICDs were obtained from each mouse sample for fluorescence intensity analysis and statistical analysis. GraphPad Prism 8.0.1 software (San Diego, CA, USA) was used for the statistical analyses. Statistical analyses between two groups were conducted using two-tailed unpaired or paired Student’s t-test. Comparisons between >two groups were performed using one-way ANOVA. Statistical significance was set at P <0.05. All statistical analyses are indicated in the corresponding figure legends.

**14. Western Blot**

Total cellular protein was extracted from heart tissue and HL-1 cells using RIPA lysis buffer (Beyotime) containing a protease inhibitor cocktail. The samples were subsequently separated using 12.5% SDS-PAGE and transferred onto nitrocellulose membranes. The membranes were then incubated with the following primary antibodies: Biotin-labeled goat anti-mouse Igκ (1:1000 SouthernBiotech), rabbit anti-mouse Cx43 (1:1000; Proteintech), rabbit anti-mouse N-cadherin (1:1000; Proteintech), rabbit anti-desmoplakin (1:1000, Proteintech), GAPDH (1:1000, ZSGB-Bio) and β-actin (1:1000, ZSGB-Bio). The secondary antibodies conjugated with HRP (ZSGB-Bio) were applied at a concentration of 1:10,000 and visualized using with ECL chemiluminescence (GE). Western Blots were quantified by densitometry using ImageJ software.

**15. Generation of conditional cardiac-specific Igκ-knockout mice**

To generate inducible cardiac-specific Igκ cKO mice, transgenic mice expressing a tamoxifen-inducible Cre recombinase protein under the control of the α-myosin heavy chain promoter, αMHC/MerCreMer mice (MCM) were intercrossed with Igκ^flox/flox^ mice (obtained from Shanghai Biomodel Organism Science & Technology Development Co., Ltd) in a C57BL/6 genetic background. To induce Cre recombination, 8- to 12-week-old male αMHC/MerCreMer; Igκ^flox/flox^ (Cre^+^; Igκ^f/f)^ mice were treated with 80mg/kg of tamoxifen (Sigma) via intraperitoneal injection once a day for 4 consecutive days. αMHC-Cre^-^; Igκ^f/f^ mice and αMHC-Cre^+^ mice with tamoxifen age- and sex-matched littermates were utilized as control mice. Animal housing conditions and all experimental procedures were conducted in accordance with the Institutional Animal Care and Use Committee of China.

**16. Construction of adenovirus**

The adeno-associated virus serotype 9 vector-cTnT-Cre (AAV9-cTnT-Cre) was produced by WZ Biosciences. 4x10^11^vg/g AAV9-cTnT-Cre were injected intravenously into 8-week-old Igκ^f/f^ mice and WT mice. Four weeks after injection, the mice were harvested for relevant detection.

The full-length cDNA of mouse IgκV17-121/J2/C was inserted into the adeno-associated virus serotype 9 vector-cTnT (AAV9-cTnT) to construct the AAV9-cTnT-Igκ virus (produced by Likeli, Beijing, China). 5x10^11^vg/g of AAV9-cTnT-Igκ or AAV9-CMV-GFP was injected intravenously into 10-week-old cardiac Igκ knockout mice (αMHC-Cre^+^; Igκ^f/f)^, whereas the same volume of AAV9-CMV-GFP was injected intravenously into αMHC-Cre^+^ mice. Four weeks after injection, tamoxifen was injected to induce Igκ gene knock down. Western Blot of myocardial tissues was performed to confirm the re-expression efficiency.

**17. Systolic blood pressure analysis**

Systolic blood pressure was measured in conscious mice by using a BP-98A Blood Pressure Analysis System (Softtron). Briefly, animals were placed in a plastic chamber maintained at 37°C, and a cuff with a pneumatic pulse sensor was attached to the tail. We measured the systolic blood pressure five times per animal and recorded the mean overall blood pressure.

**18. Echocardiography analysis**

To evaluate left ventricular function and dimension of mice heart, transthoracic two-dimensional echocardiography was performed on mice sedated with 1.5% isoflurane using a Visual Sonics Vevo 770 ultrasound system (Visual Sonics) equipped with a 17.5-MHz linear array transducer. M-mode tracings in the parasternal short axis view were used to measure left ventricular posterior wall thicknesses (LVPW) and left ventricular inner dimension (LVID) at end-diastole, which were used to calculate left ventricular fractional shortening (FS) and the ejection fraction (EF) according to standard formulas.

**19. Sarcomere shortening analysis**

Isolated cardiomyocytes were placed in the experimental chamber of an inverted microscope (Olympus) with permanent perfusion of Tyrode’s solution containing 500μM Ca^2+^ heated to 37°C. Myocytes were electrically stimulated (15V, 0.5 Hz) and sarcomere shortening was detected using a video-based detection system (IonOptix) on intact myocytes. Six twitches per myocyte were collected for each sample (control mice, n =11; cKO mice, n=6). Signal-averaged data were analyzed to determine resting sarcomere length, peak shortening normalized for resting sarcomere length (percent peak height), and time to peak shortening (TTP).

**20. Electrocardiography analysis**

Surface electrocardiogram recordings were performed on mice anaesthetized with 1–1.5% pentobarbital sodium. The needle electrodes were used and placed in the conventional lead II position. A differential amplifier amplified the signals in the bandwidth of 0.1–1000 Hz, and the signals were filtered using an adaptive 60 Hz filter. The signals were digitized at 3000 Hz, and analysed using electrocardiogram Analysis Software (QRS phenotyping, Calgary, Canada).

**21. Electron microscope analysis**

The Heart tissues fixed with 3% glutaraldehyde were chopped into 1 mm^2^ × 1 mm pieces. Then, the tissues were then rinsed, dehydrated, embedded, sliced, stained, and subsequently investigated under a transmission electron microscope.

**22. Statistical analysis**

All data are presented as mean ± SD and were analyzed using Prism GraphPad software v8.0. Statistical analysis of the two experimental groups was performed using unpaired Student’s t-test. Comparisons of more than two groups were performed using one-way analysis of variance (ANOVA), and comparisons of two groups with different time points were performed using two-way ANOVA. The effect size was calculated using SPSS, and the power was calculated using the OpenEpi and PASS software. Hierarchical analysis was performed using Stata SE software. Statistical significance was set at *P* < 0.05. (* *P*<0.05, ** *P* <0.01, *** *P* <0.001, **** *P* <0.0001)

**Supporting tables**

**Table S1.** **Primer sequences**

| **Gene name** |  | **Primer sequence** | **Product size(bp)** |
| --- | --- | --- | --- |
| Igκ constant region | Sense primer | GGGACCAAGYTGGARATVAA | 256 |
|  | Antisense primer | CTCGTCCTTGGTCAACGTG |  |
| Igκ variable region | Sense primer | GAYATTGTGMTSACMCARWCTMCA | 311 |
|  | Enternal antisense primer | GTCCTGATCAGTCCAACTGTTCAG |  |
|  | Internal antisense primer | AGNTTGGTTCCACCRCCGAACG |  |
| CD20 | Sense primer | TTCAAACTTCCAAGCCGTATG | 241 |
|  | Enternal antisense primer | GAGTTTAAGGAGCGATCTC |  |
|  | Internal antisense primer | GACAGCAGAACCACATTAGAT |  |
| Myh6 | Sense primer | AACGTGTCATCCAGTACTTTG | 242 |
|  | Antisense primer | CAGAAGGTAGGTCTCTATGTCTG |  |
| Myh7 | Sense primer | GATTATCCAGTGGAACATTAGG | 162 |
|  | Antisense primer | GACTTCTCTAGTGCATCTTTGAC |  |
| cTnI | Sense primer | ACGTGGAAGCAAAAGTCACC | 189 |
|  | Antisense primer | CCTTCTTCACCTGCTTGAGG |  |
| cTnT | Sense primer | GAGGAACAGGAAGAGCAAGA | 191 |
|  | Antisense primer | ACCAAGTTGGGCATGAAGAG |  |
| GAPDH | Sense primer | CAAGGTCATCCATGACAACTTTG | 498 |
|  | Antisense primer | GTCCACCACCCTGTTGCTGTAG |  |

**Table S2. Barcode information**

|  | **barcode-F** | **barcode-R** |
| --- | --- | --- |
| 1 | atcacg | atgagc |
| 2 | cgatgt | attcct |
| 3 | ttaggc | caaaag |
| 4 | tgacca | caacta |
| 5 | acagtg | caccgg |
| 6 | gccaat | cacgat |
| 7 | cagatc | cactca |
| 8 | acttga | caggcg |
| 9 | gatcag | catggc |
| 10 | tagctt | catttt |
| 11 | agtcaa | ctagct |
| 12 | agttcc | ctatac |
| 13 | atgtca | ctcaga |
| 14 | ccgtcc | gacgac |
| 15 | gtagag | taatcg |
| 16 | gtccgc | tacagc |
| 17 | gtgaaa | tataat |
| 18 | gtggcc | tcattc |
| 19 | gagtgg | tcggca |
| 20 | ggtagc | atcacg |

**Table S3.** **The information of Antibodies.**

| **Antibody** | **Supplier name** | **Catalog number** | **Lot number** | **Dilution ratio** |
| --- | --- | --- | --- | --- |
| Rabbit anti-mouse/human Igκ | Proteintech | 14678-1-AP | 00048685 | IF: 1:100 |
| Rabbit anti-mouse α-actinin | Proteintech | 11313-2-AP | 00025160 | IF: 1:100 |
| Rabbit anti-mouse Cx43 | Proteintech | 26980-1-AP | 00049951 | IF: 1:100/WB: 1:1000 |
| Rabbit anti-mouse N-cadherin | Proteintech | 22018-1-AP | 00059228 | IF: 1:100/WB: 1:1000 |
| Guinea pig anti-mouse/human Plakoglobin | Progen | GP57 | 610051A | IF: 1:100 |
| Rabbit anti-mouse/huma PKG | Proteintech | 27872-1-AP |  | IF: 1:100/WB: 1:1000 |
| Mouse anti human Plakophilin 2 | Abcam | Ab151402 |  |  |
| Rabbit anti-mouse/human Desmoplakin | Proteintech | 25318-1-AP | 00053324 | IF: 1:100/WB: 1:1000 |
| Rabbit anti-mouse/human DSG2 | Abcam | ab150372 |  | IF: 1:100 |
| Rabbit anti-mouse/human DSG2 | Proteintech | 21880-1-AP |  | WB: 1:1000 |
| Goat anti-mouse/human Desmoplakin | Sigma | SAB2501972 | 10151P1 | IF:1:50 |
| Guinea pig anti-Plectin | Progen | BP5115 | 606241-1 | IF: 1:100 |
| Rabbit anti-Plectin | Proteintech | 29170-1-AP |  | IF: 1:100/WB: 1:1000 |
| Mouse anti-Desmin eFluor 660 | eBioscience | 50-9747 | E25134-101 | IF: 1:100 |
| Mouse anti-Myosin Heavy Chain Alexa Fluor 488 | eBioscience | 53-6503 | E13425-106 | IF: 1:100 |
| TNNT1 Monoclonal antibody | Proteintech | 68631-1-Ig |  | IF:1:100 |
| Myc Tag polyclonal antibody | Proteintech | 16286-1-AP |  | WB: 1:1000 |
| GFP Tag polyclonal antibody | Proteintech | 50430-2-AP |  | WB: 1:1000 |
| Alpha Actin polyclonal antibody | Proteintech | 23660-1-AP | 00053839 | IF: 1:100 |
| Actin-Tracker Red-Rhodamine | Beyotime | C2207S |  | IF: 1:200 |
| Mouse anti-GAPDH | ZSGB-BIO | TA-08 | 200041012 | WB: 1:1000 |
| Mouse anti-β-actin | ZSGB-BIO | TA-09 | 19AW0505 | WB: 1:1000 |
| Goat anti-Rabbit IgG Alexa Fluor 488 | Abcam | Ab150077 | GR3203087-1 | IF: 1:1000 |
| Donkey anti-Goat TRITC | Jackson Immuno Research | 705-025-003 | 124902 | IF: 1:200 |
| Goat anti-Guinea pig IgG Alexa Fluor 594 | Abcam | Ab150188 | GR306964-4 | IF: 1:1000 |
| Goat anti-Rabbit IgG Alexa Fluor 568 | Invitrogen | A11011 | 1842719 | IF: 1:1000 |
| Donkey anti mouse IgG Alexa Fluor 488 | Invitrogen | A21202 | 2229195 | IF: 1:1000 |
| Donkey anti rabbit IgG Alexa Fluor 488 | Invitrogen | A21206 | 2256732 | IF: 1:1000 |
| Donkey anti mouse IgG Alexa Fluor 568 | Abcam | Ab175472 | GR3173739-2 | IF: 1:1000 |
| Donkey anti goat IgG Alexa Fluor 488 | Abcam | Ab150129 | GR3198891-1 | IF: 1:1000 |
| Goat anti-Guinea pig IgG Alexa Fluor 647 | Abcam | Ab150187 | GR295133-3 | IF: 1:1000 |

**Table S4. Antibodies used in Immunofluorescence.**

| Figure | Molecule | Primary Antibody | | | Secondary Antibody | | |
| --- | --- | --- | --- | --- | --- | --- | --- |
|  |  | Antibody | Catalog | Dilution | Antibody | Catalog | Dilution |
| Fig1B | Igκ | Rabbit anti-mouse/human Igκ | 14678-1-AP | 1:100 | Goat anti-Rabbit IgG Alexa Fluor 488 | Ab150077 | 1:1000 |
|  | Plakoglobin | Guinea pig anti-mouse/human PKG | GP57 | 1:100 | Goat anti-Guinea pig IgG Alexa Fluor 594 | Ab150188 | 1:1000 |
| Fig1D | Igκ | Rabbit anti-mouse/human Igκ | 14678-1-AP | 1:100 | Goat anti-Rabbit IgG Alexa Fluor 568 | A11011 | 1:1000 |
| Fig1H | Igκ | Rabbit anti-mouse/human Igκ | 14678-1-AP | 1:100 | Goat anti-Rabbit IgG Alexa Fluor 488 | Ab150077 | 1:1000 |
|  | Plakoglobin | Guinea pig anti-mouse/human PKG | GP57 | 1:100 | Goat anti-Guinea pig IgG Alexa Fluor 594 | Ab150188 | 1:1000 |
| sFig1A | α-actinin | Rabbit anti-human/mouse α-actinin | 11313-2-AP | 1:100 | Goat anti-Rabbit IgG Alexa Fluor 488 | Ab150077 | 1:1000 |
|  | TNT2 | Mouse anti-human/mouse TNT | 68631-1-Ig | 1:100 | Donkey anti mouse IgG Alexa Fluor 568 | Ab175472 | 1:1000 |
| sFig1C | Igκ | Rabbit anti-mouse Igκ | 14678-1-AP | 1:100 | Goat anti-Rabbit IgG Alexa Fluor 488 | Ab150077 | 1:1000 |
|  | Plakoglobin | Guinea pig anti-mouse/human PKG | GP57 | 1:100 | Goat anti-Guinea pig IgG Alexa Fluor 594 | Ab150188 | 1:1000 |
| sFig1E | Igκ | Rabbit anti-mouse Igκ | 14678-1-AP | 1:100 | Goat anti-Rabbit IgG Alexa Fluor 568 | A11011 | 1:1000 |
|  | Myosin | Mouse anti-Myosin Alexa Fluor 488 | 53-6503 | 1:100 |  |  |  |
| sFig1H | Myosin | Mouse anti-Myosin Alexa Fluor 488 | 53-6503 | 1:100 |  |  |  |
| sFig2D | Igκ | Rabbit anti-mouse Igκ | 14678-1-AP | 1:100 | Goat anti-Rabbit IgG Alexa Fluor 488 | Ab150077 | 1:1000 |
|  | Plakoglobin | Guinea pig anti-mouse/human PKG | GP57 | 1:100 | Goat anti-Guinea pig IgG Alexa Fluor 594 | Ab150188 | 1:1000 |
| Fig3B | Igκ | Rabbit anti-mouse/human Igκ | 14678-1-AP | 1:100 | Goat anti-Rabbit IgG Alexa Fluor 568 | A11011 | 1:1000 |
| Fig3C | N-cadherin | Rabbit anti-mouse N-cadherin | 22018-1-AP | 1:100 | Goat anti-Rabbit IgG Alexa Fluor 488 | Ab150077 | 1:1000 |
|  | Cx43 | Rabbit anti-mouse Cx43 | 26980-1-AP | 1:100 | Goat anti-Rabbit IgG Alexa Fluor 568 | A11011 | 1:1000 |
| Fig3E | Plakoglobin | Guinea pig anti-mouse/human PKG | GP57 | 1:100 | Goat anti-Guinea pig IgG Alexa Fluor 594 | Ab150188 | 1:1000 |
| Fig3F | Desmoplakin | Rabbit anti-mouse DSP | 25318-1-AP | 1:100 | Goat anti-Rabbit IgG Alexa Fluor 568 | A11011 | 1:1000 |
| sFig3B | N-cadherin | Rabbit anti-mouse N-cadherin | 22018-1-AP | 1:100 | Goat anti-Rabbit IgG Alexa Fluor 488 | Ab150077 | 1:1000 |
|  | Cx43 | Rabbit anti-mouse Cx43 | 26980-1-AP | 1:100 | Goat anti-Rabbit IgG Alexa Fluor 488 | Ab150077 | 1:1000 |
| sFig3C | N-cadherin | Rabbit anti-mouse N-cadherin | 22018-1-AP | 1:100 | Goat anti-Rabbit IgG Alexa Fluor 488 | Ab150077 | 1:1000 |
|  | Cx43 | Rabbit anti-mouse Cx43 | 26980-1-AP | 1:100 | Goat anti-Rabbit IgG Alexa Fluor 488 | Ab150077 | 1:1000 |
|  | Plakoglobin | Guinea pig anti-mouse/human PKG | GP57 | 1:100 | Goat anti-Guinea pig IgG Alexa Fluor 594 | Ab150188 | 1:1000 |
| sFig3D | Plakoglobin | Guinea pig anti-mouse/human PKG | GP57 | 1:100 | Goat anti-Guinea pig IgG Alexa Fluor 594 | Ab150188 | 1:1000 |
| sFig3E | Desmoplakin | Rabbit anti-mouse DSP | 25318-1-AP | 1:100 | Goat anti-Rabbit IgG Alexa Fluor 568 | A11011 | 1:1000 |
| sFig3F | DSG | Rabbit anti-mouse DSG2 | ab150372 | 1:100 | Goat anti-Rabbit IgG Alexa Fluor 568 | A11011 | 1:1000 |
| sFig3G | α-actinin | Rabbit anti-mouse α-actinin | 11313-2-AP | 1:100 | Goat anti-Rabbit IgG Alexa Fluor 568 | A11011 | 1:1000 |
| Fig4C | Igκ | Rabbit anti-mouse/human Igκ | 14678-1-AP | 1:100 | Goat anti-Rabbit IgG Alexa Fluor 488 | Ab150077 | 1:1000 |
|  | Desmoplakin | Goat anti-mouse DSP | SAB2501972 | 1:50 | Donkey anti-Goat TRITC | 705-025-003 | 1:200 |
| Fig4D | Desmoplakin | Rabbit anti-mouse DSP | 25318-1-AP | 1:100 | Goat anti-Rabbit IgG Alexa Fluor 488 | Ab150077 | 1:1000 |
|  | N-cadherin | Rabbit anti-mouse N-cadherin | 22018-1-AP | 1:100 | Goat anti-Rabbit IgG Alexa Fluor 488 | Ab150077 | 1:1000 |
|  | Cx43 | Rabbit anti-mouse Cx43 | 26980-1-AP | 1:100 | Goat anti-Rabbit IgG Alexa Fluor 488 | Ab150077 | 1:1000 |
| sFig4A | N-cadherin | Rabbit anti-mouse N-cadherin | 22018-1-AP | 1:100 | Goat anti-Rabbit IgG Alexa Fluor 488 | Ab150077 | 1:1000 |
|  | Cx43 | Rabbit anti-mouse Cx43 | 26980-1-AP | 1:100 | Goat anti-Rabbit IgG Alexa Fluor 488 | Ab150077 | 1:1000 |
|  | Plakoglobin | Guinea pig anti-mouse/human PKG | GP57 | 1:100 | Goat anti-Guinea pig IgG Alexa Fluor 594 | Ab150188 | 1:1000 |
| Fig5C | DSP | Rabbit anti-mouse DSP | GP57 | 1:100 | Goat anti-Rabbit IgG Alexa Fluor 568 | Ab150188 | 1:1000 |
|  | N-cadherin | Rabbit anti-mouse N-cadherin | 22018-1-AP | 1:100 | Goat anti-Rabbit IgG Alexa Fluor 568 | A11011 | 1:1000 |
|  | Cx43 | Rabbit anti-mouse Cx43 | 26980-1-AP | 1:100 | Goat anti-Rabbit IgG Alexa Fluor 568 | A11011 | 1:1000 |
| sFig5A | Igκ | Rabbit anti-mouse/human Igκ | 14678-1-AP | 1:100 | Goat anti-Rabbit IgG Alexa Fluor 488 | Ab150077 | 1:1000 |
|  | Desmin | Mouse anti-Desmin eFluor 660 | 50-9747 | 1:100 |  |  |  |
|  | F-actin | Actin-Tracker Red-Rhodamine | C2207S | 1:200 |  |  |  |
| sFig5B | Igκ | Rabbit anti-mouse/human Igκ | 14678-1-AP | 1:100 | Goat anti-Rabbit IgG Alexa Fluor 488 | Ab150077 | 1:1000 |
|  | Plectin | Guinea pig anti-mouse/human Plectin | BP5115 | 1:100 | Goat anti-Guinea pig IgG Alexa Fluor 594 | Ab150188 | 1:1000 |
| Fig6A | Igκ | Rabbit anti-mouse/human Igκ | 14678-1-AP | 1:100 | Goat anti-Rabbit IgG Alexa Fluor 488 | Ab150077 | 1:1000 |
|  | Desmin | Mouse anti-Desmin eFluor 660 | 50-9747 | 1:100 |  |  |  |
|  | Actin | Alpha Actin polyclonal antibody | 23660-1-AP | 1:100 | Goat anti-Rabbit IgG Alexa Fluor 568 | A11011 | 1:1000 |
| Fig6B | Desmin | Mouse anti-Desmin eFluor 660 | 50-9747 | 1:100 |  |  |  |
|  | F-actin | Actin-Tracker Red-Rhodamine | C2207S | 1:200 |  |  |  |
| Fig6C | Igκ | Rabbit anti-mouse/human Igκ | 14678-1-AP | 1:100 | Goat anti-Rabbit IgG Alexa Fluor 488 | Ab150077 | 1:1000 |
|  | Plectin | Guinea pig anti-mouse/human Plectin | BP5115 | 1:100 | Goat anti-Guinea pig IgG Alexa Fluor 594 | Ab150188 | 1:1000 |
| Fig6D | Igκ | Rabbit anti-mouse/human Igκ | 14678-1-AP | 1:100 | Goat anti-Rabbit IgG Alexa Fluor 488 | Ab150077 | 1:1000 |
|  | Plectin | Guinea pig anti-mouse/human Plectin | BP5115 | 1:100 | Goat anti-Guinea pig IgG Alexa Fluor 594 | Ab150188 | 1:1000 |

**Supplement figures**

**Figure S1. Igκ expression and transcript in cardiomyocyte.**

**A.** TNT2 and a-actinin staining was used as CM marker in hiPSC-CM. Scale bar =20 μm. B. Igκ staining was found to be colocalized with PKG on the cell membrane of hiPSC-CMs.Pearson's Correlation=0.8443. Igκ, immunoglobulin kappa light chain; PKG, plakoglobin.

**C.** Igκ colocalized with PKG in CM. Scale bar =25 μm. Pearson’s Correlation Rate=0.5811. PKG, plakoglobin; CM, cardiomyocytes; **D.** Igκ positive signal was found on the ICD of CM of WT mice by immuno-electron microscope. The scale bar is 500 nm for the representative image and 200nm for the zoomed-in images. **E.** Igκ staining (red) was detected on the cross striation of primary cultured CM of neonatal mice. Myosin(green) was used to identify CM. Scale bar =100 μm. **F.** Single CM was picked by a mouth pipette under microscopy and schematic overview of the single-cell sequencing workflow in CM . **G**. Transcripts of Igκ was detected in single CM. Heart of WT mice was used as positive control. Transcripts of CD20 was detected in single CM. Spleen of WT mice was used as positive control. Transcripts of cTnT was detected in single CM. Heart tissue of WT mice was used as positive control. CM, cardiomyocytes. **H.** Isolated CM of neonatal mice. Myosin was used to identify CM. Scale bar =20 μm. CM, cardiomyocytes. **I**. Transcripts of Igκ variable region and constant region in CM and FB by RT-PCR. CM, cardiomyocytes; FB, fibroblast. **J.** Igκ knockdown on mRNA levels in HL-1 cells by two independent siRNA and Igκ transcript was detected by RT-PCR. **K.** Sequence of Igκ J region to C region which was amplified in **I and J. L.** Sequence of Igκ variable region in single CM, FB and HL-1.

**Figure S2.** **Igκ transcript was found in cardiomyocyte.**

**A.** Genotype of control and cKO mice were identified by PCR. Primers were designed on both sides of flox region, the product size was 432bp when Igκ gene was deleted. **B.** Igκ expression on mRNA level in control and cKO mouse heart tissue by RT-PCR. **C.** Igκ expression on protein level in control and cKO mouse heart tissue by WB. **D.** Colocalization of Igκ (green) and PKG (red) on the ICD in control and cKO heart tissue. DAPI (blue) was used to stain nuclei. Scale bar =50 μm. Igκ, immunoglobulin kappa light chain; PKG, plakoglobin. **E.** P-R duration and R-R duration of cKO mice compared with control mice by ECG. **F.** P-R duration, R-R duration and Heart rate of cKO mice compared with control mice treated with ISO by ECG assay. **G.** Igκ knockdown on protein levels in hiPSC-CM cells by two independent siRNA. GAPDH was used as an internal control. The protein level of Igκ are normalized to β-actin loading control. 3 repeated experiments have been made. Quantitative data were shown as means ± SD. Statistical analysis in **E** and **F** were performed with unpaired t-test. Statistical analysis in **G** was performed with one-way ANOVA, followed by Tukey’s multiple comparisons test. * *P*<0.05, ** *P* <0.01, *** *P* <0.001, **** *P* <0.0001.

**Figure S3****. Transgenic Igκ deficiency led to structural abnormalities of ICDs.**

**A.** The width of ICD was measured below. n=6-19 ICD from 3 mice per group. Two-way ANOVA with Sidak’s multiple comparisons test (P<0.0001, F=34.593, η^2^_partial_=0.706). **B.** N-cadherin and Cx43 staining on the ICD in both control and cKO mice for consecutive four days after tamoxifen injection by confocal (green). Cx43, connexin 43. Scale bar = 25 μm**. C.** Colocalization of N-cadherin and PKG on the ICD of cKO and control mice for consecutive four days after tamoxifen injection by confocal. Colocalization of Cx43 and plakoglobin on the ICD of cKO and control mice for consecutive four days after tamoxifen injection by confocal. Scale bar =25 μm. Cx43, connexin 43; PKG, plakoglobin. **D.** PKG staining on the ICD in both control and cKO mice for consecutive four days after tamoxifen injection by confocal (red). Scale bar = 25 μm. PKG, plakoglobin. E. DSP staining on the ICD in both control and cKO mice for consecutive four days after tamoxifen injection by confocal (red). Scale bar = 25 μm. DSP, desmoplakin. F. DSG staining on the ICD in both control and cKO mice for consecutive four days after tamoxifen injection by confocal (red) and STED (red). he scale bar is 25 μm for the representative image and 2.5 μm for the zoomed-in images. DSG, desmoglein. G. α-actinin staining on the CM in both control and cKO mice for consecutive four days after tamoxifen injection. Quantitative data were shown as means±SD. * *P*<0.05, ** *P* <0.01, *** *P* <0.001, **** *P* <0.0001.

**Figure S4. Colocalization of N-cadherin, Cx43 and Plakoglobin on the ICD of cKOmice, control mice and cKO mice injected with AAV9-cTnT-Igκ.**

**A.** Colocalization of N-cadherin and PKG on the ICD of cKO mice, control mice and cKO mice injected with AAV9-cTnT-Igκ (Rescue mice). Colocalization of Cx43 and PKG on the ICD of cKO mice, control mice and Rescue mice. Scale bar = 25 μm. Cx43, connexin 43; PKG, plakoglobin.

**Figure S5. Protein-protein interaction domain between Igκ and plectin.**

**A.** Immunofluorescence staining shows the colocalization of Igκ with desmin and actin in HL-1 cells. Two specific siRNA for Igκ was used to knock down Igκ in HL-1 cells. Igκ, immunoglobulin kappa light chain. B**.** Immunofluorescence staining for Igκ and plectin on the cell junction of HL-1 cells. Two specific siRNA for Igκ was used to knock down Igκ in HL-1 cells. One-way ANOVA followed by Dunnet’s multiple comparisons test (Igκ:*P*<0.0001, F=76.349, η^2^_partial_=0.911; Plectin: *Pa*<0.0001, F=79.332, η^2^_partial_=0.914). **C.** Construction of Igκ variable region, constant region, full-length of Igκ and plectin-C terminal region plasmid. **D.** PlectinC-GFP vector and Igκ vectors, including variable region (Igκ-V-Myc), constant region (Igκ-C-Myc) and entire Igκ ( Igκ-FL-Myc) were co-transfected and co-expressed in 293T cells, the interaction of plectin-C and Igκ was analysed by co-IP assays. **E.** Igκ knock down by two specific siRNA and treated with CHX for 0h, 4h, 8h,12h and the mRNA level of plectin was measured by real-time PCR. **F.** Line chart of the data of **E**. Two-way ANOVA with Sidak’s multiple comparisons test. Data represent means±SD,

* *P* <0.05, ** *P* <0.01, *** *P* <0.001, **** *P* <0.0001.

1. W.C. Claycomb, N.A. Lanson, Jr., B.S. Stallworth, et al.,“HL-1 cells: a cardiac muscle cell line that contracts and retains phenotypic characteristics of the adult cardiomyocyte,*” Proc Natl Acad Sci U S A* 95 no.6 (1998): 2979-84.

2. L. Sala, B.J. van Meer, L.G.J. Tertoolen, et al.,“MUSCLEMOTION: A Versatile Open Software Tool to Quantify Cardiomyocyte and Cardiac Muscle Contraction In Vitro and In Vivo,*” Circ Res* 122 no.3 (2018): e5-e16.

3. Z. Shi, Q. Zhang, H. Yan, et al.,“More than one antibody of individual B cells revealed by single-cell immune profiling,*” Cell Discov* 5 (2019): 64.

4. F. Tang, C. Barbacioru, E. Nordman, et al.,“RNA-Seq analysis to capture the transcriptome landscape of a single cell,*” Nat Protoc* 5 no.3 (2010): 516-35.
